# Modulating the Structure and Composition of Single‐Atom Electrocatalysts for CO_2_ reduction

**DOI:** 10.1002/advs.202304424

**Published:** 2023-12-03

**Authors:** Weiren Chen, Xixiong Jin, Lingxia Zhang, Lianzhou Wang, Jianlin Shi

**Affiliations:** ^1^ Shanghai Institute of Ceramics Chinese Academy of Sciences 1295 Dingxi Road Shanghai 200050 P. R. China; ^2^ Center of Materials Science and Optoelectronics Engineering University of Chinese Academy of Sciences 19A Yuquan Road Beijing 100049 P. R. China; ^3^ School of Chemistry and Materials Science Hangzhou Institute for Advanced Study University of Chinese Academy of Sciences 1 Sub‐lane Xiangshan Hangzhou 310024 P. R. China; ^4^ Nanomaterials Centre School of Chemical Engineering and Australian Institute for Bioengineering and Nanotechnology The University of Queensland St Lucia QLD 4072 Australia

**Keywords:** coordination environment, electrochemical CO_2_ reductions, electronic structures, product selectivity, single‐atom catalysts, support effects

## Abstract

Electrochemical CO_2_ reduction reaction (eCO_2_RR) is a promising strategy to achieve carbon cycling by converting CO_2_ into value‐added products under mild reaction conditions. Recently, single‐atom catalysts (SACs) have shown enormous potential in eCO_2_RR due to their high utilization of metal atoms and flexible coordination structures. In this work, the recent progress in SACs for eCO_2_RR is outlined, with detailed discussions on the interaction between active sites and CO_2_, especially the adsorption/activation behavior of CO_2_ and the effects of the electronic structure of SACs on eCO_2_RR. Three perspectives form the starting point: 1) Important factors of SACs for eCO_2_RR; 2) Typical SACs for eCO_2_RR; 3) eCO_2_RR toward valuable products. First, how different modification strategies can change the electronic structure of SACs to improve catalytic performance is discussed; Second, SACs with diverse supports and how supports assist active sites to undergo catalytic reaction are introduced; Finally, according to various valuable products from eCO_2_RR, the reaction mechanism and measures which can be taken to improve the selectivity of eCO_2_RR are discussed. Hopefully_,_ this work can provide a comprehensive understanding of SACs for eCO_2_RR and spark innovative design and modification ideas to develop highly efficient SACs for CO_2_ conversion to various valuable fuels/chemicals.

## Graphical abstract

First, a thorough description of the key factors of SACs for eCO_2_RR and the corresponding modification strategies for metal atom centers, coordination structure, and electronic properties. Depending on the strategies, changes in the adsorption and desorption behavior of CO_2_ and intermediates subsequently occur to affect the catalytic performance of SACs. The effects of SACs on eCO_2_RR brought by different supports, including carbons, organic frameworks, metals, and oxides, are summarized. The reaction mechanisms of SACs on eCO_2_RR are discussed based on the variety of products, such as CO, HCOOH, CH_4_, CH_3_OH, and C_2+_ chemicals (e.g., C_2_H_4_, C_2_H_5_OH). Finally, the remaining challenges in this promising research field are raised, followed by a perspective on future research directions.

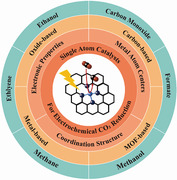

## 1 Introduction

With the ever‐increasing CO_2_ emission from massive consumption of fossil fuels, there is an urgent need to mitigate the greenhouse gas toward sustainable development.^[^ ^1^ ^]^ Currently, there are three plausible strategies to convert such excessive CO_2_ emissions: 1) direct reduction of CO_2_; 2) CO_2_ capture and storage (CCS); and 3) conversion and utilization of CO_2_.^[^ ^2^ ^]^ Compared to approaches 1 and 2, converting CO_2_ into highly value‐added products not only reduces CO_2_ emission but also creates profits at the same time, achieving bifunctional net‐zero emissions. However, it is difficult to break the stable linear centrosymmetric structure of CO_2_ unless under harsh conditions such as high temperature, high pressure, or high overpotential.^[^ ^3^ ^]^

Among the strategies of CO_2_ utilization, electrochemical CO_2_ reduction reaction (eCO_2_RR) shows good potential for its ability to activate CO_2_ molecules and produce value‐added products under mild conditions using renewable electricity. eCO_2_RR is a thermodynamically uphill process involving multi‐electron transfer.^[^ ^4^ ^]^ Nitopi et al. concluded equations for eCO_2_RR involved different numbers of electrons (*x*CO_2_ + *n*H^+^ + ne^−^ → product + *y*H_2_O), which decide the variety of final products.^[^ ^5^ ^]^ To meet the practical demands, it is necessary to improve the selectivity and current density of eCO_2_RR at low overpotentials, hence developing highly efficient electrocatalysts with flexible electronic structures and various adsorption behaviors is vital for eCO_2_RR.

Single‐atom catalysts (SACs) have recently exhibited great catalytic performances on various reactions due to their high atomic utilization efficiency and flexible metal coordination environments.^[^ ^6^ ^]^ When loaded on conductive substrates such as carbons and metals, SACs are suitable for electrocatalytic reactions.^[^ ^7^ ^]^ In contrast to bulk metal electrodes or metal nanoparticles (NPs), each center metal atom of SACs can participate in the catalytic reaction, hence maximizing the contacts between catalysts and reactants.^[^ ^8^ ^]^ In the field of eCO_2_RR, SACs exhibit high selectivity to simple C_1_ products (CO, formate) due to their high atomic utilization and homogeneous active sites, but they are not efficient in completing the complex reaction process involved in CH_3_OH, CH_4_, and C_2+_ products, which makes their application limited. Therefore, new strategies should be developed to overcome these limitations in designing and synthesizing SACs, promoting their application in the field of eCO_2_RR. This can come from other catalysts, supports, or experimental conditions. Recent works show that regulating the geometric and electronic structures of SACs is the key to improving their intrinsic activity, especially in boosting the conversion rate of CO_2_ to highly value‐added C_2+_ products. Hence, we believe that this field deserves a comprehensive and timely review to summarize recent development of SACs and the future prospects for eCO_2_RR application.

Previous reviews on this topic mostly focused on the performance and synthesis of SACs, or CO as the main product.^[^ ^9^ ^]^ Nevertheless, other important factors and possible products are ignored. In this Review, focusing on electronic structure modification, we summarize a series of modification schemes for SACs. Moreover, to expand our horizon on the application of SACs in the future, we have added two more unique parts in our review, further summarizing the current SACs from the perspectives of supports (carbons, organic frameworks, etc.) and products (CO, formate, CH_4_, etc.). The influences of supports and electronic structure on the selectivity and activity of SACs for eCO_2_RR are discussed. Unlike the previous reviews, the synthesis methods of SACs are thoroughly discussed in each section. Apart from the above contents, the role of in situ or ex situ characterizations is also deeply discussed in this review, stating the geometric structure evolution and electronic structure adjustment of center active sites during eCO_2_RR on SACs, which further influences the adsorption and desorption behavior of reactants, intermediates, and products, as well as the reaction energy barrier under various conditions. Through the overall discourse structure of this review, the structure‐performance relationship of SACs for eCO_2_RR is clearly presented. Furthermore, we provide the fundamental understanding and generic ideas to guide the design of SACs for eCO_2_RR to achieve highly value‐added products.

**Scheme 1:**
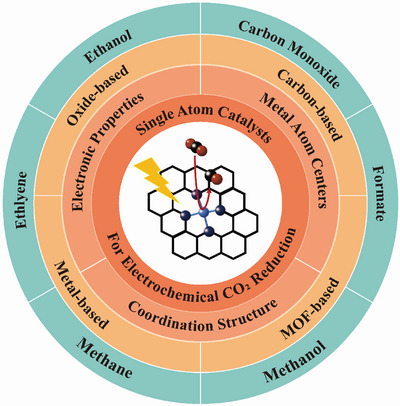
Schematic illustration of research hotspots including prominent features, reaction mechanisms, typical material systems, and main products of SACs for eCO_2_RR.

Based on the above considerations, this review includes the following contents: First, we give a thorough description of the key factors of SACs for eCO_2_RR and the corresponding modification strategies for metal atom centers, coordination structure, and electronic properties in Section 2. Depending on the strategies, changes in the adsorption and desorption behavior of CO_2_ and intermediates subsequently occur to affect the catalytic performance of SACs. In Section 3, the effects of SACs on eCO_2_RR brought by different supports, including carbons, organic frameworks, metals, and oxides, are summarized. The reaction mechanisms of SACs on eCO_2_RR are discussed based on the variety of products, such as CO, HCOOH, CH_4_, CH_3_OH, and C_2+_ chemicals (e.g., C_2_H_4_, C_2_H_5_OH) in Section 4 (**Scheme** **1**). Finally, in Section 5, the remaining challenges in this promising research field are raised, followed by a perspective on future research directions.

## 2 Key Factors of SACs for eCO_2_RR

Due to the homogenous active sites in SACs, key factors including the atomic variety of central active sites, and coordination environments (related to supports and heteroatom doping on them, etc.) influence the catalytic performance of SACs. Among them, the support changes of SACs will lead to a series of variations such as coordination environments and electronic structures, so this review will focus on the application of SACs in eCO_2_RR under different supports in Section 3. In this Section, we will introduce metal‐nitrogen‐carbon (M─N─C: single‐atom sites supported on carbon substrate and coordinated with nitrogen atoms) catalysts, the most extensively investigated SACs. It is worth mentioning that the central active sites of different SACs can change their intrinsic activity, which makes it possible that the product selectivity in eCO_2_RR can switch between CO, formate, or other value‐added chemicals. Increasing or decreasing the coordination number, changing coordination atoms, or doping heteroatoms may break the symmetric structure of the original M‐N_4_ sites, causing changes in the electronic structure or microstructure of the center active sites (e.g., bond length), thereby further improving the reactivity of SACs to a specific product. **Table** **1**. listed some typical SACs reported in recent years.

**Table 1:** Classification and summary of typical SACs.

Catalysts	Structure of active sites	Support	Synthesis method	Measurement conditions	Main products	FE	Current density [mA·cm^−2^]	Onset potential [V vs RHE]	Stability [h]	Reference
NiSA‐NGA‐900	Ni─N_4_	N‐doped graphene oxide aerogel	impregnation‐pyrolysis	0.5 m KHCO_3_ H‐cell	CO	90.2%	−8	−0.8	6	[18]
NiNP CNRs	Ni─N_4_ Ni NPs	N‐doped carbon nanorods	pyrolysis‐etching	0.5 m KHCO_3_ H‐cell	syngas	/	/	/	/	[20]
CBNNiGb‐700	Ni─N_4_	N‐doped graphene	pyrolysis‐etching	1 m KOH Flow‐cell	CO	97%	−308	−0.5	100	[36]
Ni_1_‐NSC	Ni─N_3_S_1_	N, S‐doped carbon	pyrolysis	MEA	CO	> 99%	−320	/	200	[23]
Co─N_5_/HNPCSs	Co─N_5_	hollow N‐doped porous carbon spheres	molecular catalyst anchoring	0.2 m NaHCO_3_ H‐cell	CO	99.4%	−4.5	−0.4	10	[25c]
Co‐CNTs‐MW	Co─N_x_ (*x* < 4)	carbon nanotubes	impregnation‐pyrolysis	MEA	CO	> 95%	−350	−0.4	24	[28]
Co─HNC	Co─C_2_N_2_	N‐doped 3D hollow carbon structure	impregnation‐pyrolysis	0.1 m KHCO_3_ H‐cell	syngas	/	/	/	/	[27]
Fe─N─C	Fe─N_4_	N‐doped carbon	impregnation‐pyrolysis	0.5 m KHCO_3_ H‐cell	CO	93.5%	−18	−0.3	/	[30]
Bi SAs/NC	Bi─N_4_	N‐doped carbon	MOF assisted method‐pyrolysis	0.1 m NaHCO_3_ H‐cell	CO	97%	−3.9	−0.4	4	[14b]
Zn SAs/N─C	Zn─N_4_	N‐doped carbon nanofibers	pyrolysis	Flow‐cell	CO	94.7%	−121.5	−0.7	30	[37]
(Cl, N)‐Mn/G	Mn─N_4_Cl	N‐doped carbon	pyrolysis	0.5 m KHCO_3_ H‐cell	CO	97%	−10	−0.4	12	[38]
CdN_4_S_1_/CN	Cd─N_4_S_1_	N‐doped carbon nanosheets	pyrolysis	0.5 m [Bmim]PF_6_ in MeCN solution H‐cell	CO	99.7%	−182.2	/	24	[39]
Ag_1_/MnO_2_	Ag─O	MnO_2_	surface reconstruction	0.5 m KHCO_3_ H‐cell	CO	95.7%	−3.4	−0.9	9	[12b]
MAF‐2E	Cu─Cu	cuprous triazolate frameworks	MOF assisted method	0.1 m KHCO_3_ H‐cell	C_2_H_4_	51.2%	−10	−1.0	10	[40]
NiPc‐COF	Ni─N_4_	COF	molecular catalyst anchoring	0.5 m KHCO_3_ H‐cell	CO	98%	−35	−0.6	10	[41]
Ni‐CTF	Ni─N_4_	CTF	molecular catalyst anchoring	H‐cell	CO	90%	−2.4	−0.48	/	[42]
In‐SAs/NC	In─N_4_	N‐doped carbon	impregnation‐pyrolysis	0.5 m KHCO_3_ H‐cell	formate	96%	−8.87	−0.4	60	[13a]
Sn^δ+^‐N─C	Sn^δ+^─N Sn^δ+^─C	N‐doped graphene	freeze‐vacuum drying‐pyrolysis	0.25 m KHCO_3_ H‐cell	formate	74.3%	−11.7	−0.96	200	[13c]
Sb SA/NC	Sb^δ+^─N_4_	N‐doped carbon nanosheets	pyrolysis‐etching	0.5 m KHCO_3_ H‐cell	formate	94%	−2.5	−0.8	10	[13d]
Mo@NG	Mo─O	N‐doped graphene	pyrolysis	4 mol% [Emim]BF_4_ solution H‐cell	formate	≈27%	/	0.0	7.8	[43]
NiSn‐APC	N_4_─Ni─Sn‐N_4_	hierarchical integrated carbon nanosheet array	impregnation‐pyrolysis	0.5 m KHCO_3_ H‐cell	formate	86.1%	−43.7	−0.6	23	[44]
np─Cu_1_Au	Cu─O	Au	dealloying method	0.1 m KHCO_3_ H‐cell	CO	> 90%	−15.4	−0.4	40	[45]
CoPc‐NH_2_/CNT	Co─N_4_	carbon nanotubes	molecular catalyst anchoring	0.1 m KHCO_3_ H‐cell	CH_3_OH	44%	−10.2	−0.82	5	[32a]
CuSAs/TCNFs	Cu─N_4_	through‐hole carbon nanofibers	impregnation‐electrospinning‐pyrolysis	0.1 m KHCO_3_ H‐cell	CH_3_OH	44%	−93	−0.4	50	[46]
SA─Cu─MXene	Cu─O_3_	MXene	MXene assisted method	0.1 m KHCO_3_ H‐cell	CH_3_OH	59.1%	−21.3	−0.8	30	[47]
Cu/p‐Al_2_O_3_	Cu─O	Al_2_O_3_	electrostatic interaction	Flow‐cell	CH_4_	62%	−153	−0.6	/	[48]
SA/Zn‐MNC	Zn─N_4_	microporous N‐doped carbon	pyrolysis	1 m KHCO_3_ H‐cell	CH_4_	85%	−31.8	−1.26	35	[49]
Cu‐CDs	Cu─N_2_O_2_	carbon dots	pyrolysis	0.5 m KHCO_3_ H‐cell	CH_4_	78%	40	−0.8	6	[50]
Cu─N─C‐800 Cu─N─C‐900	Cu─N_2_ Cu─N_4_	N‐doped carbon	pyrolysis	0.1 m KHCO_3_ H‐cell	CH_4_ C_2_H_4_	38.6% 24.8%	−14.8 −6.84	−0.7	10	[51]
Cu/C‐0.4	Cu─O Cu_3_	carbon	pyrolysis	0.1 m KHCO_3_ H‐cell	C_2_H_5_OH	91%	−2.1	−0.8	16	[35b]
Cu_0.5_NC	Cu─N_4_ Cu─Cu	N‐doped carbon	impregnation‐pyrolysis	0.1 m CsHCO_3_ H‐cell	C_2_H_5_OH	55%	−16.2	−0.8	/	[35a]

### 2.1 Metal Atom Centers

Products of eCO_2_RR on metal bulk electrodes in aqueous electrolytes were summarized by Hori: 1) Pb, Hg, Tl, In, Cd, and Bi tend to produce HCOO^−^; 2) Au, Ag, Zn, Pd, and Ga yield CO; 3) Cu and its alloy enable the production of CO, HCOO^−^, and even hydrocarbons and oxygenates.^[^ ^10^ ^]^ Similarly, as the size of metals decreases to the scale of atomic dispersion or single atom, the variety of center metal atoms in SACs plays a crucial role in the catalytic performance of eCO_2_RR due to their unique electronic structures or local environments, resulting in dramatic differences in product distribution, Faradic efficiency, and current density.^[^ ^9^, ^11^ ^]^ Although noble metal Au and Ag catalysts exhibit good activity in eCO_2_RR, few researchers have discussed single Au or Ag atom catalysts for eCO_2_RR in consideration of the ability of other low‐price metals to demonstrate superior performance.^[^ ^12^ ^]^ For instance, main group metals (e.g., In, Sn, Sb) prefer to produce formate.^[^ ^13^ ^]^ However, when the size of the main group element Bi is reduced to a single‐atom scale, it could be more inclined to generate CO during eCO_2_RR.^[^ ^14^ ^]^ In addition, a Bi/Zn dual SAC developed by Meng and co‐workers realized an adjustable CO/H_2_ ratio from 0.20 to 2.92.^[^ ^15^ ^]^ It was simulated by theoretical calculations that alkali metal atoms (Li, Na, etc.) supported on graphdiyne incline to form a strong bond with *OCHO intermediate (*: adsorption species) which is beneficial for the conversion of CO_2_‐to‐HCOOH.^[^ ^16^ ^]^ Nowadays, *3d* transition metal elements (e.g., Fe, Co, Ni, Cu, Zn) have been most widely used as center atoms in SACs for eCO_2_RR, on which CO is the main product.

Ni single‐atom sites in SACs are highly attractive on account of their superior selectivity for CO_2_‐to‐CO.^[^ ^17^ ^]^ An impregnation‐pyrolysis method was conducted to prepare a Ni SAC (NiSA‐NGA) with graphene oxide (GO) as support. Rich defects in GO sheets assisted in trapping Ni ions. On the optimized NiSA‐NGA catalyst, the Faradic efficiency (FE) of CO reached 90.2% at −0.6 V versus RHE (Reversible Hydrogen Electrode), and the Tafel slope was 125 mV·dec^−1^, indicating the rate‐determining step (RDS) is the formation of *COOH intermediate.^[^ ^18^ ^]^ The researchers put forward the possible steps during the conversion of CO_2_‐to‐CO: 1) the adsorbed CO_2_ molecule accepts a couple of electron and proton to generate *COOH intermediate; 2) *COOH intermediate transforms to *CO intermediate after combining with another couple of electron and proton; 3) *CO desorbed on catalyst surface to produce CO. Particularly, bulk Ni metal tend to be considered as HER active sites due to strong *CO binding affinity. However, when the size of bulk Ni metal decreases to single atoms, the production distribution can transfer from H_2_ to CO.^[^ ^19^ ^]^ Adopting this characteristic, Zhu et al. attempted to control the ratio of Ni single‐atom sites and Ni nanoparticle sites through acid leaching, a suitable syngas ratio could be obtained from 1:9 to 19:1.^[^ ^20^ ^]^ In order to achieve eCO_2_RR at high current density, it is no longer sufficient to conduct experiments solely in the H‐cell with a small working area and low current density. Researchers have attempted to conduct relevant experiments in flow‐cell and membrane electrode assembly (MEA) to reach the goal. Flow‐cells can not only increase the working area of the cathode but also adopt alkaline electrolytes as the medium for reaction. The incoming CO_2_ gas and alkaline electrolytes are separated through the cathode gas diffusion layer, and the reaction between CO_2_ and alkaline electrolytes is suppressed. In general, current density can be greatly improved in flow‐cell. Ni‐SAC@NCs synthesized by Guo and co‐workers reached a CO partial current density of −187.7 mA·cm^−^ in a flow‐cell with 1 M KHCO_3_ at 2.7 V cell voltage, while the current density in H‐cell was below −40 mA·cm^−2^.^[^ ^21^ ^]^

Furthermore, due to the zero‐gap design which reduces ohmic resistance, MEA enables higher efficiency. Two sides of the membrane will direct contact with the cathode and anode catalysts. During operation, humidified CO_2_ is supplied to the cathode side without flowing electrolyte.^[^ ^22^ ^]^ For instance, Chen et al. took S atoms to replace one coordinated N atom in Ni─N_4_ sites, then the obtained Ni_1_‐NSC could achieve a FE(CO) over 99% with a high current density of −225 mA·cm^−2^ in MEA.^[^ ^23^ ^]^

**Figure 1:**
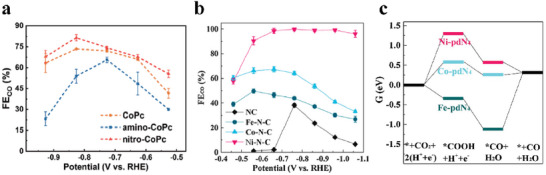
a) FE(CO) of various CoPc samples at different applied potentials.^[^ ^26^ ^]^ Copyright 2020, Springer Nature. b) FE(CO) of M─N─C (M = Fe, Co, Ni) and NC at different applied potentials; c) Free energy diagram for the conversion of CO_2_ to CO on M‐pdN_4_.^[^ ^24^ ^]^ Copyright 2021, Elsevier.

Co single‐atom sites have a moderate *COOH intermediate formation energy and *CO desorption energy.^[^ ^24^ ^]^ Immobilizing Co phthalocyanine (CoPc) molecules on certain supports is a facile way to prepare Co SACs.^[^ ^25^ ^]^ Ren et al. prepared a CoPc/G catalyst and further introduced nitro ligands or amino ligands onto CoPc molecules to obtain nitro‐CoPc/G and amino‐CoPc/G, respectively. Among the three catalysts, nitro‐CoPc/G had the highest performance with an FE(CO) of 85.4% at −0.83 V versus RHE. Whereas amino‐CoPc/G showed poorer performance than CoPc/G (**Figure** **1**a). This difference probably resulted from the electron‐withdrawing ability of the nitro ligands and the electron‐donating ability of the amino ligands.^[^ ^26^ ^]^ Except for CO_2_‐to‐CO, Co SACs sometimes can also produce syngas. Co‐HNC incorporating bifunctional Co and pyridinic‐N sites. followed a dual‐sites mechanism. As the center Co atoms were poisoned by KSCN^−^, coordinated pyridinic‐N sites functioned as HER sites and increased H_2_ production. According to density functional theory (DFT) calculation, C atoms in CO_2_ molecules tended to bond with Co atoms, while two O atoms were more likely to bond with N atoms in Co‐HNC, showing that the center Co atoms are the active sites of eCO_2_RR. The sum evolution rate of CO_2_ and H_2_ achieved 425 mmol g^−1^·h^−1^ at −1.0 V versus RHE with an ideal (CO/H_2_) ratio of 1/2.^[^ ^27^ ^]^ Sun et al. synthesized a Co SAC (Ni‐CNTs‐MW) through a microwave‐assisted strategy, which realized a current density of −350 and −200 mA·cm^−2^ in flow‐cell and MEA with a FE(CO) of over 95%, respectively.^[^ ^28^ ^]^

Fe single atoms are also introduced to SACs for eCO_2_RR.^[^ ^29^ ^]^ However, the *CO intermediate on them has relatively high desorption energy, which impedes the conversion of CO_2_‐to‐CO. Hence, a synergetic effect between Fe─N_4_ sites and the defects (e.g., nanopores) of support benefiting eCO_2_RR was proposed. In aid of nanopores, the binding strength of *COOH and *CO intermediates on the catalyst is balanced, thus *CO poisoning can be avoided and CO was more easily desorbed from Fe single‐atom sites.^[^ ^30^ ^]^In another case, graphene support with rich pore edges could modulate the local electronic structure of Fe single‐atom sites. At −0.58 V versus RHE, the obtained Fe─N─G‐p achieved a FE(CO) of 94%, 13% higher than that on the common graphene‐supported catalyst. The pore structure could promote the electrochemical active surface area (ECSA) and the accessibility of active sites. Theoretical calculations prove that Fe─N_4_‐pore and Fe─N_4_‐edge sites shift down the d band center of Fe single‐atom sites and provide a longer Fe─C bond when *CO intermediate is generated so that CO molecules are easier to desorb.^[^ ^31^ ^]^ This case proved the possibility of taking advantage support effect to promote the performance of the SACs.

SACs with Fe, Co, and Ni single‐atom centers are often taken into account simultaneously.^[^ ^32^ ^]^ Wang et al. prepared three SACs of M─N─C (M referred to as Fe, Co, Ni). Electrochemical measurement in an H‐cell exhibited that FE(CO) was in the order of Ni─N─C > Co─N─C > Fe─N─C, manifesting that Ni─N─C had the excellent selectivity of CO_2_‐to‐CO with the maximum FE(CO) of 99.8% (Figure 1b). However, Co─N─C displayed the highest CO turnover frequency (TOF) and CO partial current density at a wide range of potentials. Co─N─C also processed the ability to produce syngas and the ratio of CO/H_2_ could be regulated from 0.5 to 2.11. Theoretical calculations disclosed that the rate‐limiting steps of eCO_2_RR on Ni‐pdN_4_ and Fe─pdN_4_ (pd referred to metal atoms coordinated by four pyridine N atoms in the carbon matrix) sites were *COOH formation and *CO desorption, respectively, while Co─N─C had moderate *COOH formation energy and *CO desorption energy (Figure 1c). Meanwhile, Ni─N_4_ sites demonstrated the largest gap of thermodynamic limiting potentials between eCO_2_RR and HER, explaining the reason why Ni─N─C had the best selectivity of CO_2_‐to‐CO.^[^ ^24^ ^]^ The calculated density of states (DOS) of the central metal atoms in the M─N─C SAC demonstrated that only the central Ni atom in Ni─N─C showed the upward and downward symmetric peaks, indicating the absence of unpaired electrons in the outermost *d* orbital of Ni. The presence of unpaired electrons in the outermost *d* orbit of the central Fe or Co atom is confirmed in the other M─N─C SACs. Differential charge density (DCD) calculation displayed that Ni─N─C had the highest electron cloud density around the central Ni atoms, further demonstrating that Ni─N─C was most favorable for electron transfer and eCO_2_RR to occur. This difference in electronic structure also made Ni─N─C the catalyst with the highest eCO_2_RR activity among the three catalysts.^[^ ^33^ ^]^

Like the function of Cu electrodes which enable 16 products during eCO_2_RR, Cu SACs also exhibit great possibility in the field of eCO_2_RR. Yang et al. reported a scalable one‐pot thermal activation strategy to produce a Cu SAC. The final catalyst Cu SAs/NC had a lower onset potential of −0.23 V versus RHE and performed an FE(CO) of 92% at −0.7 V versus RHE. Furthermore, researchers attempted to expand its production scale and successfully produced 22.8 g of Cu SAs/NC in one batch.^[^ ^34^ ^]^ Additionally, Cu sites can produce not only CO but also C_2+_ products because Cu‐based catalysts are the only reliable materials that enable the realization of C─C coupling during eCO_2_RR up to now. Thus, several Cu SACs have been synthesized to achieve this goal.^[^ ^35^ ^]^ However, the highly dispersed Cu single‐atom sites seriously hinder the progress in C─C coupling. To discuss this phenomenon in more detail, the research concerning C_2+_ product evolution on Cu SACs will be presented in the later part.

### 2.2 Coordination Structure

N‐doped carbon (N─C) supports can anchor atomically isolated metal atoms, and usually, the number of coordinated nitrogen atoms is four to prevent atom aggregation. Up to now, most SACs for eCO_2_RR are in the form of M─N─C. The strong interaction between metal center atoms and supports leads to the residual charge on the center metal atom so that the intrinsic activity will be promoted to enhance reactant activation.^[^ ^52^ ^]^ Benefiting from the special structure, the coordination environment of the active sites can be easily adjusted. In this part, the impacts of coordination structure change on the performance of SACs are discussed.

#### 2.2.1 Coordinated Number

As for N─C‐based SACs, a suitable local N coordination environment should be emphasized, and tuning the coordination number is the most straightforward way to modulate the electron distribution of metal center atoms to improve catalytic performance.^[^ ^53^ ^]^ A Cu─N─C catalyst achieves an excellent FE(CO) of 99% at −0.67 V versus RHE and a large CO partial density of −131.1 mA·cm^−2^ at −1.17 V versus RHE. Every active site of the as‐prepared Cu─N─C catalyst contains one Cu─N_3_ structure and three proton‐saturated N atoms. The hydrogen bond formed between the O atom of *COOH intermediate and the nearby H atom in the H‐saturated N atom is more beneficial for the formation of *COOH intermediate than the conventional Cu─N_4_ site. After such a process of proton transfer, *CO is more likely to be desorbed from the Cu atom to generate the final product CO.^[^ ^54^ ^]^ As metal ionic liquids (MILs) were precursors, through the hydrogen bonds between anions and cations in MILs, it was possible to control the coordination number of the metal atom centers. After the pyrolysis of the precursors containing [Bmim]_2_[CuCl_4_] and CNTs, the final SAC with Cu─N_3_ sites attained a FE(CO) of over 90% in a wide potential range from −0.42 to −0.92 V versus RHE.^[^ ^55^ ^]^

Changing calcination temperature seems a workable method to modulate the coordination environment. Under increased temperature, M─X (X = N, O, or other coordinated atom) bond can be broken, and more electrons will transfer from the carbon substrate to the metal center atoms, contributing to the improved activity of catalysts. This phenomenon was investigated by Rong and co‐workers based on XPS O 1s spectra. A N/O mixed coordination catalyst (Ni─N_3_O) was first prepared at 500 °C. Further temperature elevation would oblige the coordinated O atom to leave the catalyst. The obtained vacancy‐defect catalyst (Ni─N_3_‐V) performed a high FE(CO) of over 94% at −0.8 V versus RHE and a current density of −65 mA·cm^−2^ in an H‐cell containing CO_2_‐saturated 0.5 m KHCO_3_, while the catalyst Ni─N_4_ without vacancy merely reached a FE(CO) of 85% at −0.8 V versus RHE under the same experimental conditions.^[^ ^56^ ^]^ Cheng et al. utilized microwave‐exfoliated graphene oxide (MEGO) with rich defects to support Ni single atoms under 800 °C. According to the Fourier‐transformed extended X‐ray absorption fine structure (EXAFS) spectra, Ni─N─MEGO possessed a more complicated coordination environment than Ni phthalocyanine (NiPc). DFT calculation demonstrated that the Ni─N_3_ moieties anchored on the edge of pore defects of MEGO had superior performance on eCO_2_RR.^[^ ^57^ ^]^

**Figure 2:**
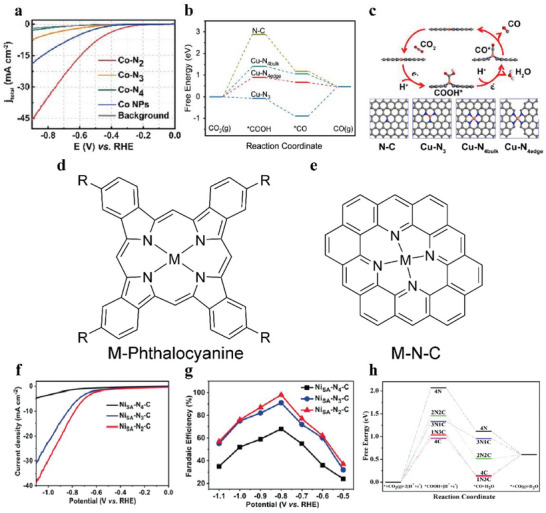
a) LSV curves of Co─N_2_, Co─N_3_, Co─N_4_, Co NPs, and pure carbon paper as background in an H‐cell with CO_2_‐saturated 0.5 m KHCO_3_ as electrolyte.^[^ ^58^ ^]^ Copyright 2018, Wiley‐VCH. b) Free energy diagrams of eCO_2_RR on N─C and Cu─N_x_; c) The reaction pathways and optimized catalyst models of N─C and Cu─N_x_ (Gray, blue, and orange represent the C, N, and Cu atoms, respectively). Copyright 2018, Elsevier. d) Structure of M‐Phthalocyanine; e) Structure of M─N─C. f) LSV curves of NiSA‐N_x_‐C in CO_2_‐saturated 0.5 m KHCO_3_ electrolyte; g) FE(CO) of NiSA‐N_x_‐C at varied potentials.^[^ ^66b^ ^]^ Copyright 2020, Wiley‐VCH. h) Free‐energy diagram of CO_2_ reduction to CO on the five complexes: NiN_4_(4N), NiN_3_C(3N1C), NiN_2_C_2_(2N2C), NiNC_3_(1N3C), and NiC_4_(4C).^[^ ^66a^ ^]^ Copyright 2020, Wiley‐VCH.

When the coordination number is <4, the unsaturated coordination structure will weaken the restraining ability of carbon substrate to the metal atom active sites for CO_2_ molecule adsorption and desorption. During this process, center metal atoms are sometimes slightly removed from the carbon substrate plane, resulting in the extension of the M─N bond, which is advantageous for charge transfer and CO_2_ activation. An unsaturated coordination SAC (Co─N_2_) was synthesized and exhibited the most positive onset overpotential of 110 mV relative to Co─N_3_ and Co─N_4_ SACs, and exhibited the lowest charge resistance beneficial for electron transfer (**Figure** **2**a).^[^ ^58^ ^]^ Jia et al. fabricated a catalyst (SA‐NG‐NV) with Ni‐pyridinic‐N_2_V_2_ unsaturated structure through a plasma treatment strategy. The obtained catalyst achieved an FE(CO) of over 90% during a 20 h electrolysis at −0.7 V versus RHE. In contrast to those symmetrical Ni─N_4_ sites, SA‐NG‐NV displayed higher and more distinct pre‐edge peaks in local electric dipole transition according to Ni K‐edge X‐ray absorption near edge structure (XANES), indicating its local electric dipole transition and asymmetrical structure compared to the centrosymmetric structure of Ni─N_4_ moiety. XPS N 1s spectra discovered that pyrrolic N species would turn into pyridinic N species to stabilize the Ni center atom after the introduction of vacancy defects, and the length of Ni─N bonds would also be extended, which was evidenced through Fourier transform magnitudes of EXAFS spectra.^[^ ^59^ ^]^ Meanwhile, *COOH formation energy on different Ni─N sites was also identified and in the order of Ni─N_4_ > Ni─N_3_ > Ni─N_2_. This reveals that the unsaturated coordination of Ni─N_X_ is more favorable for eCO_2_RR.^[^ ^18^ ^]^ The comparison of Ni─N_x_ sites was also made by other researchers.^[^ ^59^, ^60^ ^]^ Yan et al. simulated four structures of Ni─N sites including Ni─N_4_, Ni─N_3_, Ni─N_3_V, and Ni─N_2_V_2_ (V: coordination vacancy), and found the formation energy of *COOH intermediate on the Ni─N_4_ site was higher than those on the other three unsaturated sites. The Ni─N_2_V_2_ site even had a higher formation energy of *H intermediate than *COOH intermediate, manifesting that eCO_2_RR was more competitive than HER on the Ni─N_2_V_2_ site.^[^ ^61^ ^]^

However, a converse conclusion that increasing calcination temperature led to a lower coordination number was reached on a Cu─N─C catalyst by Cheng and co‐workers. XPS N 1s and Cu 2p spectra pointed out that the peak of Cu─N would be more obvious, and the coordination number of Cu would increase with rising temperature. DFT calculations explained that Cu─N_3_ sites had a strong *CO adsorption ability resulting in a poisoning effect (Figure 2b,c).^[^ ^62^ ^]^ Similarly, Tuo et al. found that the catalyst with Fe─N_4_ sites had superior performance to those with Fe─N_3_ sites and Fe─N_2_ sites because Fe─N_4_ sites had a strong ability to suppress HER. According to the XPS N 1s spectra, the content of graphitic‐N in SA‐Fe/NG‐x (*x* = 600 800, 1000) increased with temperature elevation and was negatively correlated with the content of pyrrolic‐N.^[^ ^63^ ^]^

At present, coordination number can be characterized by EXAFS and theoretical calculation assist in explaining why different coordination number leads to different performances. Therefore, on the premise that most studies can get accurate coordination numbers, they still get the opposite conclusion. Researchers should try to focus on other factors that can impact the experimental results, such as surrounding nitrogen species content, diverse substrate carbon materials, synthesis routes, or precursors utilized.

#### 2.2.2 Coordinated Atoms

The variety of coordinated atoms is another variable to regulates the local coordination environment of single‐atom metal centers in SACs. A saturated M─N_4_ site has an analogous structure to a metal‐Pc (M─Pc) symmetrical structure. M─Pc complexes have clear independent metal active sites and coordination environment (usually planar and coordinated with four N atoms), which are similar to the common structure of SACs (M─N─C) (Figure 2d,e).^[^ ^64^ ^]^ M─Pc complexes themselves are also applied as excellent molecular catalysts in eCO_2_RR. In order to further improve their performance and stability, some researchers attempted to modify them with functional groups or anchor them on a support.^[^ ^65^ ^]^ For SACs, once the coordinated N atoms have been replaced by another atom, the original structure will suffer distortion and change into asymmetrical mode. Due to the electronegativity change of coordinated atoms, electrons will undergo redistribution and the intrinsic activity of the catalysts will be modulated. Under the synergetic effect of geometric and electronic structure changes, the performance of SACs will be flexibly adjusted.

Some researchers discovered that four Ni─N bonds gradually tended to break when the pyrolysis temperature rose to ≈800 °C, and C atoms in the substrate would participate in forming chemical bonds with Ni atoms to maintain stability.^[^ ^66^ ^]^ Gong et al. first fabricated a Ni─N─C catalyst using non‐nitrogenous MOF and Polypyrrole (PPy) molecules filling in the channels of MOF as carbon and nitrogen sources. After pyrolysis at varied temperatures, changeable coordinated Ni SACs were obtained, and their catalytic activity was in the sequence of Ni_SA_‐N_2_‐C > Ni_SA_‐N_3_‐C > NiSA‐N_4_‐C (Figure 2f–g). Ni_SA_‐N_2_‐C achieved a FE(CO) of 98% at −0.8 V versus RHE and long stability for 10 h.^[^ ^66b^ ^]^ DFT calculations manifested that Ni─N_4_ sites had relatively higher energy of *COOH formation and lower energy of *CO desorption. The N, C‐coordination of NiN_x_C_4‐x_ catalysts elevated the Ni *d* orbital position closer to the Fermi level. That was, the introduction of Ni‐C bonds caused the electron redistribution in SACs and the electron transfer among Ni─N/Ni‐C sites so that CO_2_ could be easily turned into *COOH intermediate (Figure 2h).^[^ ^66a^ ^]^ Nevertheless, when the metal atom centers were replaced by Co atoms, Geng et al. discovered that the complete N‐coordinated Co SAC had superior catalytic performance at any applied potential compared with the N, C‐coordinated counterpart. CO_2_‐temperature programmed desorption (CO_2_‐TPD) profiles and diffuse reflectance infrared Fourier transform spectroscopy (DRIFTS) spectra elucidated that CO_2_ had a higher desorption temperature, that is, a stronger binding strength on Co_1_‐N_4_ sites than Co_1_‐N_4‐x_C_x_ sites.^[^ ^67^ ^]^

**Figure 3:**
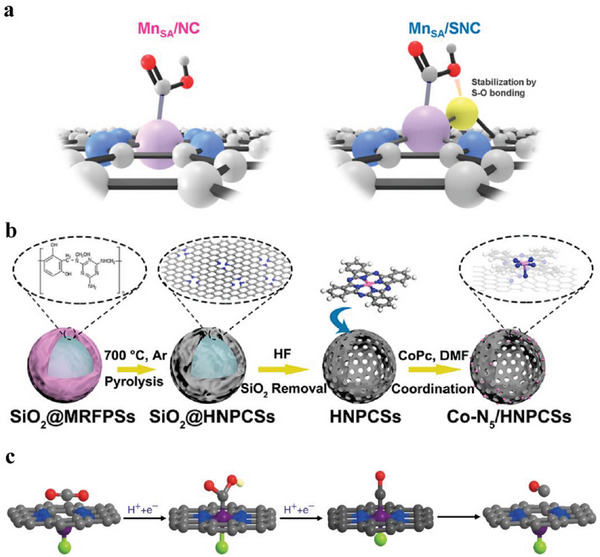
a) Schematic illustration of the intermediate *COOH adsorbed on the MnSA/NC (left side) and MnSA/SNC (right side). The white, gray, blue, red, yellow, and purple balls represent H, C, N, O, S, and Mn atoms, respectively.^[^ ^70^ ^]^ Copyright 2021, American Chemical Society. b) Schematic illustration of synthesizing Co─N_5_/HNPCSs.^[^ ^25c^ ^]^ Copyright 2018, American Chemical Society. c) Structural evolution of the active site on (Cl, N)‐Mn/G in eCO_2_RR (Mn: purple, Cl: green, N: blue, O: red, H: white, and C: gray).^[^ ^38^ ^]^ Copyright 2019, Springer Nature.

Compared with N atoms, S or P atoms have a larger diameter and lower electron negativity, enabling them to play the role of electron donor to coordinated metal atoms.^[^ ^68^ ^]^ It can be predicted that the introduction of S or P atoms will efficiently tailor the geometric and electronic structures of SACs to boost catalytic performance and reactant transport. Wang et al. chose Bi_2_S_3_ as both Bi source and S source to form an N, S‐coordinated Bi SAC, which achieved a maximum FE(CO) of 98.3% at −0.8 V versus RHE and FE(CO) of over 88% at wide range potentials, originating from the introduction of S atom and abundant channels for substance diffusion and electron transfer.^[^ ^14a^ ^]^ Li et al. synthesized a self‐standing SAC with Ni─N_3_S sites, which performed an optimal selectivity of 91% for CO without performance degradation. DFT calculations revealed that the energy barrier for the step of CO_2_RR is lower than that of HER on Ni─N_3_S sites, and the conversion of *COOH‐to‐*CO was a thermodynamic downhill process.^[^ ^69^ ^]^ An Mn SAC with Mn─N_3_S_1_ sites was fabricated by Tan et al. with an FE(CO) of ≈70% at −0.45 V versus RHE. The doped S atoms were beneficial for activating reactants and widening the limit of CO_2_ transport. According to operando X‐ray absorption spectroscopy(XAS) results, additional S─O bonds will be formed between the coordinated S atom and O atom on the OH end of *COOH intermediate due to the larger size of the S atom, leading to the stability enhancement of *COOH intermediate and the subsequent conversion of *COOH‐to‐*CO (**Figure** **3**a).^[^ ^70^ ^]^ Particularly, Jia et al. first prepared an SAC with Ni─N_2_ sites, in which the vacancy sites permit S atoms to be doped and coordinated with Ni atoms, as well as structure distortion would not happen due to the existence of vacancies. The Ni‐S bond would be broken at −0.8 V versus RHE and an S vacancy site would be generated. Ni─N_2_S sites performed a more preferable selectivity for CO_2_RR to CO than Ni─N_2_V_S_ or Ni─N_2_, and the overpotentials followed the trend of Ni─N_2_ > Ni─N_2_V_S_ > Ni─N_2_S.^[^ ^71^ ^]^ Similarly, Li et al. replaced one N atom with one P atom. The Fe─N/P‐C catalyst was obtained through pyrolyzing Fe^3+^ACB (a mixture of activated carbon black with Fe^3+^) precursor with N and P sources. Ex situ XANES spectra demonstrated that the P atom was able to stabilize the oxidation state of the Fe atom center due to its weak electronegativity, preventing Fe atoms from agglomerating into nanoparticles during eCO_2_RR. Theoretical calculations discovered that P, N‐coordinated Fe single‐atom sites could gather more electrons around Fe atoms, resulting in the relatively lower valence of Fe atoms and facilitating electron transfer and conversion of CO_2_‐to‐CO.^[^ ^72^ ^]^ Zhang et al. incorporated P and S atoms into Ga SAC (Ga‐N_4_‐C) to replace the Ga‐N bond and modulate porous carbon substrate (Ga‐N_3_S‐PC). Compared to rigid Ga‐N_4_ sites, Ga‐N_3_S‐PC obtained specific structural flexibility. In the process of electroreduction of CO_2_, this flexible 3D structure would adjust the Ga‐S and Ga‐P bonds and reduce the *COOH intermediate activation energy to generate CO. Finally, Ga‐N_3_S‐PC reached a FE(CO) of 92% at −0.3 V versus RHE in an H‐cell with 0.5 m KHCO_3_.^[^ ^73^ ^]^

Usually, the alteration of coordination atoms in M─N_4_ SACs originates from the introduction of heteroatoms during synthesis processes. Heteroatoms will attempt to replace N atoms and coordinate with the central metal atoms, adjusting the geometric and electronic structures of active sites in SACs. However, the heteroatoms introduced into supports seem to also come into the heteroatom doping or axial coordination of single‐atom sites as illustrated in the following two parts. These situations should be carefully differentiated by researchers to clarify the roles of the heteroatoms in eCO_2_RR.

#### 2.2.3 Heteroatom Doping

In this part, we will emphasize how the doped heteroatoms influence the catalytic performance of SACs. Heteroatoms doped into carbon substrates won't directly participate in the coordination, while they often bring defects and structural distortion, thus having impacts on the electron distribution of the SACs.

S is a common element used as a doped heteroatom. Chen et al. adopted the polymerization of ethylene‐dioxythiophene (EDTO) and acetonitrile in the presence of FeCl_3_ to synthesize an S, N‐doped carbon‐based Ni SAC (Ni─SN─hCNCs), which demonstrated a higher I_D_/I_G_ ratio, manifesting the existence of more defects than Ni─N─hCNCs without S dopant. S atoms dedicated electrons to Ni─N_x_ sites to promote the *d* band density, resulting in accelerated charge transfer during eCO_2_RR.^[^ ^74^ ^]^ A similar change in I_D_/I_G_ ratio was also observed by Pan et al. on an S, N‐doped carbon‐based Fe SAC (Fe─NS‐C). The charge density calculation elucidated that there were increased valence electrons on Fe atoms in S doped carbon matrix, compared with those in the common Fe─N_4_ model.^[^ ^75^ ^]^

P and F have also been incorporated into carbon substrates to adjust the electron distribution of catalysts. Sun et al. prepared a P‐doped Fe─N─C catalyst (Fe─SAC/NPC), in which P atoms were located at the third coordination shell of the Fe single atoms. The oxidation state of the center Fe atoms decreased so that more electrons could be contributed to *COOH, achieving high kinetics with a Tafel slope of 59 mV·dec^−1^.^[^ ^76^ ^]^ Han et al. decorated a Ni SAC with an F dopant (Ni─4N─C─F) and achieved a notable CO yield of 1146 mmol g_cat_^−1^·h^−1^ at −0.97 V versus RHE. In situ attenuated total reflectance infrared spectroscopy (ATR‐IR) spectra of Ni‐SAs@FNC evidenced the existence of two intense peaks assigned to C = O stretching of *COOH and the consuming H_2_O at several applied potentials. The partial density of states (PDOS) plots revealed that the shift of the *d* band center of Ni─4N─C─F toward the Fermi level made it easier to transfer electrons to the center Ni atom, leading to a slight increase of the charge density around the Ni atom.^[^ ^77^ ^]^

Based on theoretical calculations, no matter whether the heteroatoms are S, P, or F, they will all make the whole catalyst undergo electron redistribution so that increased electrons will be located at the center atom sites, which will be in favor of reactant activation and reaction progress. At present, heteroatoms doped in carbon supports of SACs are still limited to these three elements, while the difference among diverse heteroatom doping has not been unraveled. Meanwhile, the effect of the heteroatom doping content on the catalytic active site is little mentioned in this research, and this is something to be noted in future studies.

#### 2.2.4 Axial Coordination

Axial coordination extends the regulating dimension of single‐atom sites in SACs.^[^ ^78^ ^]^ It has been verified as another promising way to promote the catalytic performance of SACs by several related studies focusing on ORR or OER.^[^ ^79^ ^]^ The axial coordination strategy can be realized through two routes. The first one is to immobilize molecule catalysts on the enriched N‐doped carbon substrates. With the introduction of an axial M─N bond and the high conductivity of carbon substrates, the electron transfer rate in the whole material will be accelerated. Pan et al. supported CoPc on hollow N‐doped porous carbon spheres (HNPCSs) to form Co─N_5_ active sites (Figure 3b) and achieved a maximum FE(CO) of 99.4% at −0.79 V versus RHE.^[^ ^25c^ ^]^ Similarly, combining Fe TPP (meso‐tetraphenyl porphyrin iron (III) chloride complex) with aminated carbon nanotubes, Tuo et al. fabricated Fe─N─CNT with axially coordinated N atoms and achieved high eCO_2_RR activity and low HER reactivity.^[^ ^80^ ^]^

Nevertheless, sometimes the original axial atoms should be removed to promote eCO_2_RR performance. Miola et al. used hemin to fabricate a Fe─N─C catalyst whose original axial Fe─Cl bond was broken during heat treatment, and FE(CO) was successfully up to 99% at −0.42 V versus RHE while the value of the Fe─N─C catalyst with axial Cl coordination was only 63%.^[^ ^81^ ^]^ Furthermore, Zhang et al. put forward a two‐step pyrolysis way that connected hemin to melamine and graphene to get a Fe─N_5_ catalyst with axial Fe─N bonds and the disappearance of Cl atoms. The Fe─N_5_ catalyst showed an FE(CO) of 97% at a low overpotential of 0.35 V versus RHE.^[^ ^82^ ^]^ The above two synthetic routes proved that an appropriate coordination environment was significant for SACs and provided a feasible proposal for the conversion of bio‐derived feedstocks to SACs.

Directly forming axial bonds between metal atoms and the heteroatoms (e.g., N, O, S, F atoms) of 2D M─N─C materials is another method.^[^ ^13^, ^39^, ^76^, ^83^ ^]^ Due to the strong electron negativity of axial O atoms, Wang et al. discovered that electrons would transfer from the center metal atoms to the axial O atoms. Experiments simultaneously evidenced that Ni─N_4_─O/C had a higher Fermi level and DOS around the Fermi level was also optimized. Meanwhile, the energy barrier of the RDS, *CO_2_‐to‐*COOH, also decreased.^[^ ^84^ ^]^ Ni et al. incorporated F atoms into an Sn SAC through an indirect capturing route and constructed a C, F‐coordinated Sn‐C_2_O_2_F configuration. With the axial bonding of Sn‐F, competitive HER was suppressed and the conversion of CO_2_‐to‐HCOOH was impeded due to the highly consumed energy of convex inversion that the Sn atom was out of the carbon plane due to the axial Sn‐F bond. Thereby, Sn‐C_2_O_2_F delivered a FE(CO) of over 90% from −0.2 to −0.6 V versus RHE with a maximum current density of −186 mA·cm^−2^, showing dissimilar performance to conventional Sn‐N_4_ catalysts.^[^ ^83b^ ^]^

The performance of SACs coordinated with different axial atoms was compared by Wu and co‐workers. DFT calculations revealed that CdN_4_S_1_ had a lower energy barrier of 0.27 eV than CdN_5_ of 0.39 eV when the S atom replaced the axial N atom, which stemmed from the high spin density and charge delocalization of the S atom. Electrochemical measurement verified that CdN_4_S_1_/CN indeed achieved an astonishing FE(CO) of 99.7% at −2.4 V versus Ag/Ag^+^, yet CdN_5_/CN achieved a FE(CO) of 92.6% at the same applied potential.^[^ ^39^ ^]^

Zhang et al. prepared Mn SAC, (Cl, N)‐Mn/G, which performed the best FE(CO) of 97% at −0.6 V versus RHE. Compared to Mn^2+^Pc, the Mn─N bond was lengthened due to the axial Mn─Cl bond, which led to the structure distortion of (Cl, N)‐Mn/G. Mn K‐edge EXANES spectra showed that the valence of Mn increased when (Cl, N)‐Mn/G was immersed into CO_2_‐saturated 0.5 m KHCO_3_, while the center Mn atoms of N─Mn/G had no valence change. This phenomenon stemmed from recovering the distortion of the axial Mn─Cl bond (Figure 3c). A series of transition metal SACs axially coordinating other halogen atoms were also synthesized and demonstrated outstanding performance on eCO_2_RR.^[^ ^38^ ^]^

To sum up, eCO_2_RR performance on SACs can be effectively improved by anchoring molecule catalysts on supports or directly constructing additional axial bonds on 2D catalysts. The electron redistribution or structure distortion could change the energy barrier and the adsorption energy of intermediates. However, the axial bonds have not always improved eCO_2_RR performance and sometimes retarded the reaction.^[^ ^81^, ^85^ ^]^

### 2.3 Electronic Properties

#### 2.3.1 Oxidation State

The oxidation state of the single‐atom sites in SACs is a direct description of their electron state, which has a huge effect on the work function and the activity of SACs. Work function indicates how much energy a solid needs to accept or remove an electron, and the electron distribution of active sites can be related to their oxidation state.^[^ ^86^ ^]^ Li and co‐workers surveyed the work function of Ni single‐atoms (Ni‐SAs) and nanoparticles (Ni─NPs) on N‐doped carbon substrate via ultraviolet photoelectron spectroscopy (UPS). When the size of Ni species reduces from nanoparticles of 14.3 nm to the atomic scale, the work function decreases from 5.8 eV of 14.3 Ni NPs to 5.5 eV of Ni SAs, indicating that Ni‐SAs possess better charge transfer kinetics benefiting CO_2_ activation.^[^ ^87^ ^]^

Under most conditions, the valence of center metal atoms is between 0 of the metallic state and +n of their highest oxidation state, while their exact value of oxidation state is difficult to distinguish. XPS was carried out by Zhao and co‐workers to define the oxidation state of Ni atoms in SACs. Ni 2p_3/2_ peak was located between the Ni^0^ and Ni^2+^ peaks, unraveling that the Ni single‐atoms existed as ionic Ni^δ+^ (0 < *δ* < 2), which was in line with their XAFS results.^[^ ^88^ ^]^ A catalyst with atomically dispersed Ni^1+^ sites was developed by Yang et al. and performed an FE(CO) of 97% at an overpotential of 0.61 V and long‐time stability of 100 h. The unpaired electron in the 3d_x_^2^_‐y_^2^ orbital of Ni^1+^ sites was confirmed by the g values of 2.215 and 2.285 at room temperature and 77 K, respectively, in electron spin resonance (ESR) spectra. Operando Ni K‐edge XANES and EXAFS spectra elucidated that the Ni atomic sites would transfer electrons to the C atom of CO_2_ and display a rising oxidation state, then after eCO_2_RR, the monovalent Ni atoms would be recovered from the high oxidation state.^[^ ^89^ ^]^

**Figure 4:**
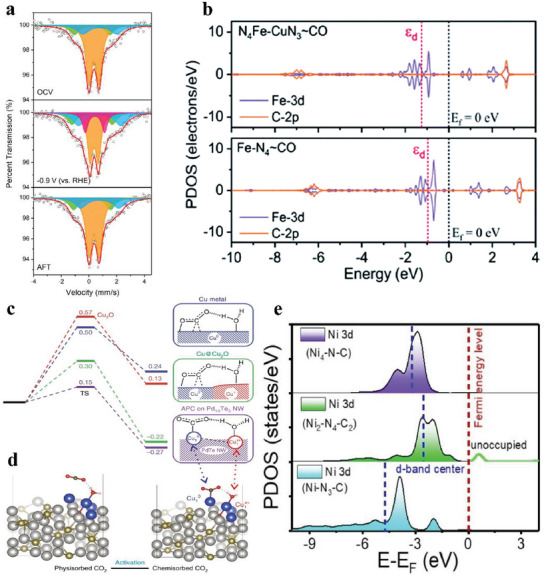
a) Operando ^57^Fe Mössbauer spectra of ^57^Fe‐enriched Fe─NC─S recorded at open circuit voltage (OCV), −0.9 V (versus RHE), and after eCO_2_RR (AFT) in CO_2_‐saturated 0.5 m KHCO_3_ solution. The orange, green, blue, and purple doublets could be assigned to LS Fe^2+^ in Fe^2+^N_4_, MS Fe^2+^ in Fe^2+^N4, HS Fe^2+^ in N─Fe^2+^N_4_, and LS Fe^2+^ in Fe^1+^N_4_, respectively.^[^ ^91^ ^]^ Copyright 2021, American Chemical Society. b) Calculated PDOS for N_4_Fe─CuN_3_ and Fe─N_4_ with *CO adsorption.^[^ ^104^ ^]^ Copyright 2020, Wiley‐VCH. c) Free energy profiles (at −0.78 V versus RHE) for CO_2_ activation on Cu, Cu@Cu_2_O, and atom‐pair catalyst (APC) of Cu_1_^0^─Cu_1_^x+^ on Pd_10_Te_3_ nanowires (TS, transition state; NW, nanowire.); d) Configurations of physisorbed CO_2_ and chemisorbed CO_2_ on Cu─APC.^[^ ^102^ ^]^ Copyright 2019, Springer Nature. e) PDOS for the Ni 3*d* orbital over Ni_2_‐N_4_‐C_2_, Ni─N_3_‐C, and Ni_4_‐N─C.^[^ ^103^ ^]^ Copyright 2022, Wiley‐VCH.

Taking Fe SACs as an example, whether the varied valences of Fe single‐atom sites show an influence on eCO_2_RR performance is worth discussing.^[^ ^90^ ^]^ Li et al. performed operando ^57^Fe Mössbauer characterization and found the appearance of low‐spin (LS) Fe^1+^N_4_ doublet in Fe─NC─S at −0.3 V versus RHE, whose content increased at more negative potentials. The content of LS Fe^2+^N_4_ doublet decreased simultaneously, indicating that Fe^+^ stemmed from Fe^2+^ and LS Fe^1+^N_4_ doublet would disappear after one eCO_2_RR cycle (**Figure** **4**a).^[^ ^91^ ^]^ In this case, in situ generated Fe^1+^ sites could be the real active sites. However, Gu et al. synthesized a Fe^3+^ SAC (Fe^3+^‐N─C) for eCO_2_RR, which performed a current density of −94 mA·cm^−2^ at −0.45 V versus RHE. The Fe K‐edge XANES spectra showed the oxidation state of Fe atoms was +3. During eCO_2_RR, the oxidation state of Fe would be reduced from +3 to +2 at −0.5 V versus RHE. As a comparison, Fe^2+^‐N─C is also prepared and presented poorer stability and inferior activity, suggesting Fe^3+^ single‐atoms were the real active sites.^[^ ^90^ ^]^ Li et al. supported Fe phthalocyanine (FePc) molecules on graphene oxide (GO) and achieved FE(CO) over 90% with a low onset potential of 190 mV. They discovered the synergistic effect of Fe^2+^Pc, Fe^3+^Pc, and GO, in which the co‐doped Fe^3+^ and Fe^2+^ single‐atom sites performed better catalytic activity than their respective sites.^[^ ^92^ ^]^

The above cases shed light on the impact of the oxidation state of single‐atom sites on the catalytic performance of SACs. Developing more methods to adjust the oxidation state of the center metal atom will be instrumental in fabricating more efficient SACs for eCO_2_RR. As for multivalent metal elements, especially Fe and Cu, the synergetic effects between single‐atom sites with different valence states provide another opportunity for developing new‐type SACs. Meanwhile, these bring a big challenge to characterize these single‐atom sites. Though several characterization technologies were applied to explore the electronic structure of the single‐atom active sites, high accuracy, and time resolution operando technologies are still highly desired.

#### 2.3.2 Dual Atom Sites

Although SACs outstand with the virtue of the highest specific surface area, high selectivity, and well‐defined active sites, it is difficult for them to exceed their intrinsic activity and regulate the product selectivity in eCO_2_RR due to the single structure of their atomic sites and the lack of synergistic effects between different sites.^[^ ^93^ ^]^ Recently, one's eyes turned to dual atom sites to construct a dual atom catalyst (DAC), which is capable of tuning the electronic structure of atomic sites and breaking the customary linear scaling relationship of intermediates while maintaining the advantages of SACs.^[^ ^94^ ^]^ For instance, Ni─N─C and Fe─N─C are two common SACs applied in eCO_2_RR, while Ni─N─C suffers from a high *COOH intermediate formation energy and Fe─N─C endures a strong bonding of *CO intermediate, blocking the reaction rate and selectivity.^[^ ^89^, ^95^ ^]^ Therefore, a dual atom sites strategy has been developed to break this linear scaling relationship. As the name implies, the dual atom sites strategy is to introduce an additional atomically dispersed active site next to the original single‐atom active site. Dual atom sites can be classified as either homonuclear or heteronuclear ones, while there is no significant difference between them, and in general, both of them introduce additional metal sites to modulate the adsorption and desorption behavior of the reactants and products on the original active sites.

Ren et al. synthesized a DAC with Ni‐Fe sites (Ni/Fe─N─C), affording a maximum FE(CO) of 98% at −0.8 V versus RHE, which surpassed Ni─N─C and Fe─N─C in a wide range of potentials due to post‐adsorption phenomenon. The mechanism of eCO_2_RR on the bimetallic Ni/Fe─N site was established to discover that strongly bonded *CO would passivate the Fe─Ni site and subsequent reduction of CO_2_ would take place on the Fe site. The binding strength of *COOH and *CO on CO‐adsorbed Ni/Fe─N─C was weakened, resulting in an accelerated catalytic process.^[^ ^96^ ^]^ Afterward, Gong et al. disclosed the decreased oxidation state of Fe atoms and increased oxidation state of Ni atoms due to the electron redistribution of Ni atom and Fe atom in a hetero‐paired catalyst (Ni/Fe─N/O‐C) through XPS Fe 2p and Ni 2p spectra. This electron redistribution made the catalyst yield a maximum FE(CO) of 99.8% at −1.5 V versus SCE (Saturated Calomel Electrode) in CO_2_‐saturated 0.5 m KHCO_3_.^[^ ^93^ ^]^

Other DACs (e.g., Cu─Fe, Ni─Zn, Ni─Co) also displayed efficient performance on eCO_2_RR.^[^ ^97^ ^]^ Feng et al. constructed a catalyst with Fe─Cu dual‐atom sites (Fe/Cu─N─C) in the structure of N_4_Fe─CuN_3_, whose FE(CO) kept over 95% at a wide potential rage from −0.4 to −1.1 V versus RHE. The dual‐atom sites moved the *d* band center closer to the Fermi level than the individual Fe─N_4_ sites according to PDOS analysis (Figure 4b), and the XANES spectra similarly confirmed that the strong electronic effect between neighboring Fe atom and Cu atom gave rise to the positive shift of the Fe K‐edge and negative shift of the Cu K‐edge.^[^ ^98^ ^]^ Zhu et al. also discovered that the introduction of Cu atoms to form Ni─Cu dual‐atom sites shifted Ni 3d orbital energy to the Fermi level. It is worth mentioning that the adsorption of *COOH intermediate involves the hybridization between the Ni 3d orbital and the C 2p orbital. Hence, the closer Ni 3d orbital energy to the Fermi level strengthened the adsorption of *COOH intermediate on the Ni atom in the Ni─Cu DAC.^[^ ^97d^ ^]^

Hu et al. introduced In atoms into the original Cu‐ZIF‐8 to anchor In─N_4_ sites beside Cu─N_4_ sites to construct a Cu─In dual atom catalyst (Cu─In─NC). The electronic donation effect originated from In─N_4_ sites and led to electron‐rich Cu active sites, strengthening the interaction between Cu sites and *COOH intermediates. Cu─In─NC demonstrated a FE(CO) of 96% at −0.7 V versus RHE in an H‐cell with 0.1 m KHCO_3_.^[^ ^99^ ^]^

Several studies focused on homogeneous dual‐atom sites.^[^ ^12^, ^100^ ^]^ For instance, Zhang et al. reported a Pd_2_ DAC with a maximum FE(CO) of 98.2% at −0.85 V versus RHE. Theoretical calculations proved that dual atom (Pd_2_) sites had the lowest barrier energy (1.25 eV) for the conversion of CO_2_‐to‐*COOH, while that of Pd_1_ sites in Pd SAC or Pdˊ_1_ sites in Pd_2_ sites was up to 1.86 eV and 1.44 eV, respectively. Compared with the Pd_1_ and Pdˊ_1_ sites, the Pd_2_ sites had the lowest oxidation state and presented the strongest Pd‐CO* bond. This suitable bonding energy between Pd_2_ sites and *CO made it easier to be broken for CO desorption.^[^ ^101^ ^]^ Jiao et al. supported a copper atom pair on Pd_4_Te_10_ alloy nanowires to synthesize a Cu atom‐pair catalyst (APC). During NaOH etching, O atoms would occupy Te atom vacancies and form special Cu_4_─O_x_ sites. One Cu atom on the surface was coordinated with oxygen atoms to form Cu^x+^, which was further bonded to another Cu atom on the surface to form a Cu^0^─Cu^x+^ atomic pair structure. In this system, Cu^0^ will adsorb one CO_2_ molecule and one H_2_O molecule will be adsorbed on Cu^x+^ to help activate the neighboring CO_2_ molecule (Figure 4c,d). This synergetic effect results in a high FE(CO) of 92% at −0.78 V versus RHE, which is 6.57 times higher than that on the pristine Pd_4_Te_10_ alloy nanowires.^[^ ^102^ ^]^ Cao et al. adopted an electrospinning method to synthesize a binuclear nickel bridging structure (Ni_2_‐N_4_‐C_2_) which is coordinated with four N atoms and two C atoms. In comparison to the traditional single‐atom site (Ni─N_3_‐C) and nanoparticle site (Ni_4_‐N─C), the conversion of *COOH to *CO and the desorption of *CO easily occur on the Ni_2_‐N_4_‐C_2_ site. According to the PDOS for the Ni 3d orbital, the d band center of the Ni_2_‐N_4_‐C_2_ site is the closest to the Fermi level, suggesting the strongest electron delocalization and promoting the electron transfer to adsorbed CO_2_ (Figure 4e).^[^ ^103^ ^]^

To have a better comparison between homogenous dual‐atom sites and heterogeneous dual‐atom sites, Zhu et al. employed DFT calculations to screen the best dual‐atom sites for eCO_2_RR. The Zn/Zn sites in Zn─N─C and the Co/Co sites in Co─N─C performed weak adsorption of *COOH intermediate, hence it was speculated that the neighboring effect between two homogeneous atoms was disadvantageous for eCO_2_RR. In contrast, in ZnCoNC, the neighboring effect aroused a downhill *CO desorption energy on Zn atoms and an uphill *COOH adsorption energy on Co atoms so that *COOH would be adsorbed on Co sites at first. Then the generated *CO intermediate on Co sites might transfer to Zn sites and subsequently be desorbed. This synergetic effect endowed ZnCoNC with a FE(CO) of 93.2% at −0.5 V versus RHE and longtime stability of 30 h.^[^ ^104^ ^]^

It is worth mentioning that Li et al. realized the conversion of CO‐to‐C_2+_ products on a Cu─Cu dual atom sites catalyst. In a flow‐cell with 0.1 m KHCO_3_, the electrochemical CO reduction reaction (eCORR) experiment reached FE(C_2_H_4_) of 32%, FE(CH_3_COOH) of 33%, and a small amount of C_2_H_5_OH and *n*‐propanol. The total FE(C_2+_) achieved was ≈ 91%.^[^ ^105^ ^]^ This work demonstrated that C─C coupling could be feasible at the Cu dual atom sites. Although this result was achieved still limited to CO electroreduction, it would provide ideas for the future design of direct conversion of CO_2_‐to‐C_2+_ products on the dual atom site catalysts. Furthermore, Shao et al. synthesized two catalysts named as BIF‐102NSs and BIF‐104 NSs. BIF‐102NSs with dimer copper (Cu_2_) units could produce C_2_H_4_, while BIF‐102NSs with single Cu units only obtained CH_4_ and CO.^[^ ^106^ ^]^ Meanwhile, Zhuo et al. probed the effect of the microenvironment of Cu─Cu sites on product distribution in cuprous triazolate frameworks. As the size of the side ligand groups in the catalyst gradually shrinks, the products on the Cu─Cu sites gradually transformed from CH_4_ to C_2_H_4_ with a maximum FE(C_2_H_4_) of 51.2%.^[^ ^40^ ^]^ This evidence demonstrated that Cu─Cu DACs have great potential for converting CO_2_ into C_2+_ products.

To sum up, DACs, derived from SACs, can be seen as consisting of two adjacent active sites compared to SACs with isolated active sites. Therefore, DACs have great opportunities to break the limitations of existing SACs. Introducing dual atom sites not only increases the metal site loading of the catalyst to enhance its activity, but also optimizes the adsorption behavior of CO_2_ and key intermediates during eCO_2_RR, and has the opportunity to regulate the catalytic reaction pathway.^[^ ^107^ ^]^ However, the products on the current DACs still point toward CO. Particularly, Cu dual atom sites can possess the ability to convert CO into C_2+_ products.^[^ ^105^ ^]^ Therefore, future DACs would focus more attention on developing or modulating dual atom sites for targeting C─C coupling during CO_2_ or CO electroreduction.

In this section, we have described several key factors of SACs, including the metal atom centers, coordination structure, and electronic properties. However, these factors change the electronic structure of active sites in SACs to adjust the catalytic performance. For example, changing the element of the atom center influences the intrinsic catalytic activity of SACs. The atom centers with different elements have diverse electronic structures, hence their selectivity for a certain product will also change accordingly. Changing the coordination environment is a more intuitive way to adjust the charge distribution in SACs, increasing/reducing the coordination number, or the involvement of heteroatoms in coordination or doping will destroy the original symmetric structure, thus causing the transfer of electrons in SACs, changing the desorption behavior of intermediates in the catalytic reaction process. The direct modification of the electronic properties of the catalyst is achieved through oxidation state regulation and dual atom sites. The presence of multiple sites makes it possible to break the linear scaling relationship of intermediates adsorption on original SACs.

## 3 Typical SACs for eCO_2_RR

Supports usually play important roles in heterogeneous catalytic reactions. The interactions between supports and active species have always been one of the pivotal themes in heterogeneous catalysis. In this section, we will introduce SACs with different supports in detail. Supports will affect the coordination environment of atomically dispersed active sites and promote the catalytic reaction process. Therefore, we will divide the introduction into four parts based on the variety of supports, including carbon‐based, organic framework‐based/derived, metal‐based, and oxide‐based SACs. We will evaluate the SACs with different supports by integrating the synthesis process and catalytic reactions and provide the advantages and disadvantages of different supports.

### 3.1 Carbon‐based SACs

Benefiting from the high conductivity and ease of being doped, carbon materials have been often used as the supports for SACs. When metal single‐atoms are anchored on carbon substrates, the strong interaction between them can stabilize the atomically dispersed metal atoms and electrons will be easy to transfer from the carbon substrates to the active sites. Carbon‐based SACs can be divided into carbon nanotubes, carbon nanosheets, and carbon nanospheres according to their morphologies.

N‐doped carbon nanotubes (CNTs) possess superior electronic conductivity and a unique tubular structure that enables fast mass diffusion, hence they are promising supports for SACs to achieve high kinetics.^[^ ^108^ ^]^ Zhao et al. fabricated a Ni SAC (NiSA‐N─CNT) through a one‐pot pyrolysis method. At elevated temperatures, Ni single‐atoms would be thermally activated, which led to internal stresses causing a curling of the layered Ni‐g‐C_3_N_4_, thereby forming a CNT structure. The as‐prepared NiSA‐N─CNT‐800 mainly produced CO and performed a TOF of 11.7 ± 0.2 s^−1^ at −0.55 V versus RHE.^[^ ^109^ ^]^ Fan et al. took Ni NPs as the growth catalyst of residual CNTs. They coated a layer of resorcinol, melamine, and formaldehyde on CNTs, and then after a pyrolysis process, Ni species were incorporated in the form of single Ni─N_3_ sites on the N‐decorated CNTs. In contrast to NC or CNTs (Ni), NC‐CNTs (Ni) possessed not only the highest ESCA but also the fastest charge transfer.^[^ ^19^ ^]^ With this method of CNTs growth, the residual Ni NPs were inevitable. Shen et al. fabricated two Ni SACs with and without acid leaching, named (Ni@NCNT/CFM(900) and H‐Ni@NCNT/CFM(900)), respectively. The FE(CO) of H‐Ni@NCNT/CFM(900) was nearly 100%, 20% higher than that of Ni@NCNT/CFM(900), indicating that the elimination of metal NPs in SACs was of vital importance. At an increased calcination temperature, it was observed that the NCNTs shell became thicker from <5 layers to >10 layers, and all Ni NPs were wrapped at the tip of NCNTs when the thickness was over 5 nm. Compared with the thinner‐walled catalyst, Ni@NCT/CFM(1000) with a thicker shell performed a FE(CO) of nearly 100%, while the H_2_ partial current density dramatically decreased, manifesting that the side reaction of HER on Ni NPs was successfully suppressed.^[^ ^110^ ^]^ Pan et al. constructed a hierarchical structure containing mesoporous carbon nanotubes and graphene nanoribbon networks (GNR). The as‐obtained catalyst Fe─N/CNT@GNR‐2 achieved a FE(CO) of 98% at −0.76 V versus RHE in an H‐cell with CO_2_‐saturated 0.1 m KHCO_3_. The hierarchically mesoporous CNT@GNR architecture provided high surface area and sufficient mass transport, meeting the demands of eCO_2_RR.^[^ ^111^ ^]^ He et al. immobilized Ni single‐ atoms on N‐doped winged carbon nanofiber (NiSA‐NWC). Abundant Ni (I) sites contributed to the delocalization of CO_2_ anti‐bond charge hence NiSA‐NWC achieved a maximum FE(CO) of over 95% at −1.6 V versus Ag/AgCl, which was 30% superior to NiNP‐NWC.^[^ ^112^ ^]^

To explore the curvature effects of support materials, Fang and co‐workers synthesized several Zn SACs using three supports: N‐doped carbon fibers (Zn SAs/N─C), carbon nanotubes (Zn SAs/CNTs) and graphene (Zn SAs/G). Zn SAs/N─C performed a maximum FE(CO) of 92.6%, while FE(CO) of Zn SAs/CNTs and Zn SAs/G only reached 82.6% and 60.1%, respectively. DFT calculation demonstrated that PD‐Zn─N_4_‐1 (PD: pyridine‐N) sites with curvature in Zn SAs/N─C performed the lowest *COOH formation energy than its counterpart without curvature. Bader charge calculation further evidenced a lower positive charge on the center Zn atoms than those in PD‐Zn─N_4_‐1 without curvature in that the *d*_x2‐y2_ orbital electrons of Zn atoms supported on nanofibers returned to Zn atoms through Zn─N bonds, resulting in negative sites with enhanced conversion of CO_2_‐to‐*COOH.^[^ ^37^ ^]^

Porous carbon nanosheets synthesized by various methods are provided with adjustable size, morphology, and pore structure and, hence are widely applied in catalytic reactions such as ORR.^[^ ^113^ ^]^ On CO_2_RR, those SACs supported on porous carbon nanosheets also performed unique performances.^[^ ^51^, ^114^ ^]^ Lu et al. attempted to anchor Ni single‐atom sites on ultrathin nanosheets through a polydopamine (PDA) assisted method. g‐C_3_N_4_ was chosen as the template, which transformed into ultrathin carbon nanosheets after pyrolysis. Their specific surface area dramatically increased from 63.5 m^2^·g^−1^ to over 1000 m^2^·g^−1^, indicating that these ultrathin N‐doped carbon nanosheets supplied more chances to contract with reactants. Consequently, the as‐prepared NiSA/N─C achieved an outstanding current density of −111.5 mA·cm^−2^ at −1.0 V versus RHE.^[^ ^115^ ^]^ Tuo et al. put forward a layered confinement reaction. After cetyltrimethylammonium bromide (CTAB) was inserted into the inter‐lamellar space of V_2_O_5_·nH_2_O xerogel, the meso‐tetra (*N*‐methyl‐4‐pyridyl) porphyrin ferric chloride (III) (FeTMPyP) group would also enter the inter‐lamellar space through ion exchange. After the subsequent thermal treatment and acid etching, a Fe SAC (MPPCN‐*x*, *x* = carbonization temperature) would be obtained. MPPCN‐750 displayed an ultrathin 2D nanosheet structure, on which rich active sites could be exposed to reactants. In a closed H‐cell with CO_2_‐saturated 0.5 m KHCO_3_ solution, MPPCN‐750 performed a superior FE(CO) of 95.9% at −0.7 V versus RHE.^[^ ^116^ ^]^

Hollow mesoporous carbon spheres (HMCS) are featured with remarkable permeability and a high specific area, thus are ideal supports for SACs.^[^ ^117^ ^]^ Yuan et al. adopted a semi‐sacrificial template method to synthesize HMCS. Ni^2+^ was immobilized on SiO_2_/polydopamine, and then high‐temperature treatment obliged Ni atomic sites to be highly dispersed on the N‐rich carbon matrix. Subsequent HF etching eliminated the SiO_2_ template and residual Ni nanoparticles. The as‐prepared SA‐Ni/N─CS gained a FE(CO) of 95.1% at −0.8 V versus RHE and the value of FE(CO) still held at 95% after 24 h of stabilization test.^[^ ^118^ ^]^ To explore the impacts of the geometrical structure of HMCS, including shell structure and pore structure, on eCO_2_RR performance, Xiong et al. designed several HMCS supports with different geometrical structures by accurately controlling the feed amount of NH_4_OH or the feed ratio of pure dopamine solution and Ni contained dopamine solution. Among all the Ni/HMCS samples, Ni/HMCS‐3‐800 incorporated by ultrathin carbon shell showed the highest BET surface area of ≈ 1220 m^2^·g^−1^ and CO_2_ adsorption capacity of ≈ 3.09 mmol·g^−1^. Compared to those samples with thicker carbon shells, the FE(CO) on Ni/HMCS‐3‐800 reached 93% at −1.1 V versus RHE. This outstanding performance could originate from the ultrathin carbon shell structure, which regulated the charge distribution and surface adsorption capacity of the catalyst. Furthermore, the pore structure of HMCS was also adjusted by changing the calcination temperature. Both Ni/HMCS‐3‐700 and Ni/HMCS‐3‐800‐FD (FD: advanced freeze‐dried precursor before calcination) with smaller pores performed relatively low current density and catalytic activity in that the small size mesopores in the carbon shell would inhibit molecule diffusion and overflow of both reactants and products.^[^ ^119^ ^]^

Up to now, self‐supported SACs have attracted increasing attention on account of their characteristics that they can be directly used as a binder‐free electrode. Zhao et al. developed a self‐diffusion method to prepare a Ni SAC (H‐CPs), which contained a 2D N─C layer and 1D N‐NCT nanotube. H‐CPs displayed excellent mechanical properties which met the demands of directly acting as electrodes without a binder. Especially, the synthesis procedures of H‐CPs were programmable and can be massively manufactured.^[^ ^120^ ^]^ Yang et al. fabricated CoSA/HCNFs with ultra‐flexibility and mechanical stability through an electrospinning method. CoSA/HCNFs contained abundant mesopores and macropores which were in favor of CO_2_ diffusion. CoSA/HCNFs could directly function as cathode after being cut into a suitable shape, which performed a high FE(CO) of 97% at −0.6 V versus RHE in an H‐cell and an outstanding CO partial current density of −211 mA·cm^−2^ at −0.9 V versus RHE in a flow‐cell. However, once CoSA/HCNFs were powdered and then drop‐casted on the electrode with the addition of Nafion solution, it demonstrated poor performance due to the sharp decline in ECSA and CO_2_ activation ability.^[^ ^121^ ^]^

**Figure 5:**
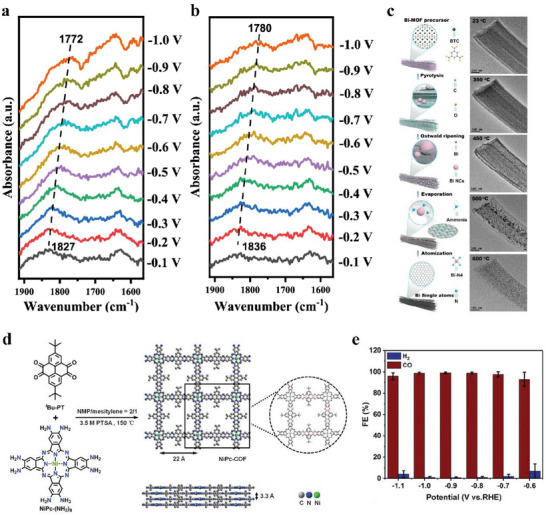
a) In situ ATR‐SEIRAS spectra of Ag_1_–N_3_/PCNC and b) Ag_1_–N_2_/PCNC.^[^ ^12c^ ^]^ Copyright 2021, American Chemical Society. c) Scheme of the transformation from Bi‐MOF to single Bi atoms and the corresponding representative in situ TEM images of Bi‐MOF pyrolyzed at different temperatures with the assistance of DCD.^[^ ^14b^ ^]^ Copyright 2019, American Chemical Society. d) Schematic illustration for the synthesis of 2D conductive NiPc‐COF with top view and side view of the slipped AA stacking structure; e) FE(CO) and FE(H_2_) from −0.6 to −1.1 V versus RHE of NiPc‐COF in CO_2_‐saturated 0.5 m KHCO_3_.^[^ ^41^ ^]^ Copyright 2020, Wiley‐VCH.

In addition to the above synthesis methods, taking advantage of MOFs to anchor metal sites or adsorb metal ions for pyrolysis to obtain carbon‐based SACs is also a commonly used method. Among the wide variety of MOFs, ZIF‐8 is the mainstream support to load doped metal atoms or precursors for MOF‐derived SACs up to now.^[^ ^46^, ^108^, ^122^ ^]^ Furthermore, Lu et al. introduced dicyandiamide (DCD) in precursors to optimize catalyst structure. As‐obtained Ni SAs/NCNTs presented bamboo‐like carbon nanotubes interweaving with irregular nanostructures. The performance of Ni/ZIF (without DCD) on eCO_2_RR was excellent at low potentials but occurred a sharp decline at −0.8 V versus RHE. However, Ni SAs/NCNTs maintained a high value of FE(CO) of ≈95% at −0.7– −1.0 V versus RHE. The introduced DCD would transform into tubular structures at elevated temperatures to trap released Ni^2+^ during the carbonization process. Therefore, Ni SAs/NCNTs provided more active sites for eCO_2_RR than Ni/ZIF, resulting in a stable value of FE(CO) at high potentials.^[^ ^123^ ^]^ Similarly, Sui et al. synthesized two Ag SACs (Ag_1_‐N_3_/PCNC and Ag_1_‐N_2_/PCNC) with and without the introduction of DCM, respectively. By comparison, Ag_1_‐N_3_/PCNC exhibited the best FE(CO) of 95% at −0.37 V versus RHE in an H‐cell with CO_2_‐saturated 0.1 m KHCO_3_ as electrolyte. In the spectra of in situ attenuated total reflectance‐surface‐enhanced infrared absorption spectroscopy (ATR‐SEIRAS), the peak assigned to linear adsorbed *CO intermediate occurred at a lower potential on Ag_1_‐N_3_/PCNC. To evidence the influence of coordination number, at a more negative potential, it was discovered that the peak of *CO intermediate on Ag_1_‐N_3_/PCNC shifted to lower wave numbers than that on Ag_1_‐N_2_/PCNC at a more negative potential, manifesting a weakened *CO intermediate adsorption on Ag_1_‐N_3_/PCNC (**Figure** **5**a,b). Hence, *CO intermediates were much easier to desorb from the active sites to form CO molecules.^[^ ^12c^ ^]^ Chen et al. introduced Fe_3_C NPs onto a common catalyst with Fe─N_4_ sites by an all‐solid ligand‐vapor method. Fe_3_C|Fe_1_N_4_ performed a smaller charger transfer resistance than Fe_1_N_4_ and a higher CO partial current density. The introduction of Fe_3_C NPs not only promoted the conductivity of the initial Fe─N_4_ catalyst but also strengthened the adsorption ability of CO_2_ on Fe─N_4_ sites and accelerated the formation of the key *COOH intermediate. In situ ATR‐FTIR spectra displayed that H_2_O was quickly consumed on Fe_3_C|Fe_1_N_4_ surface and enriched on the Fe_1_N_4_ surface, respectively, indicating the abundant *CO_2_^−^ on the Fe_3_C|Fe_1_N_4_ surface was protonated to form *COOH intermediate.^[^ ^124^ ^]^

Carbon materials, due to their facile synthesis, controllable morphology, and superior conductivity, are commonly used as supports for SACs in eCO_2_RR with high product selectivity at low applied potentials. However, under elevated applied potentials or increased current density, SACs are likely to shift from eCO_2_RR to HER, resulting in a rapid increase in FE(H_2_). Therefore, in the future design of SACs, researchers should devote to SACs capable of enduring harsh conditions with stable selectivity and long‐term stability to achieve industrial applications.

### 3.2 Organic Framework‐based SACs

As an emerging class of porous materials, metal‐organic frameworks (MOFs) are composed of two basic units: metal‐containing nodes and organic linkers, currently also possessing high surface area, tunable pore size, and adjustable internal surface properties.^[^ ^125^ ^]^ Due to the uniform distribution of metal nodes in MOFs, it is convenient to synthesize SACs or their precursors. Hou et al. concluded five virtues of MOF‐immobilized SACs: 1) Porous uniform structures and 3D repeated channels facilitate the mass transport of the substrates; 2) The structures of MOFs are easy to be finely adjusted to meet certain demands; 3) Enabling to immobilize molecule catalysts and enhancing the catalytic activation of heterogeneous catalyst without compromising their stability; 4) Conventionally introducing various metal‐based sites to achieve the synergetic interactions between different chemical components; 5) Promoting the metal loading of SACs.^[^ ^126^ ^]^ Hence, MOF‐based or derived SACs are generally used in electrochemical CO_2_RR.^[^ ^127^ ^]^

As the most used MOF, ZIF‐8 is facile to be synthesized and doped with other heteroatoms (e.g., Fe, Co, Ni, Cu) to replace Zn atoms. However, although Zn species in ZIF‐8 play a vital role in separating active sites in SACs, those Zn atoms that cannot be completely removed under pyrolysis have a negative influence on eCO_2_RR. To suppress the ineluctable Zn species in ZIF‐8 precursors, researchers attempted to adopt other MOFs without Zn species to synthesize SACs.^[^ ^14^, ^128^ ^]^ Zhang et al. adopted a solvothermal method with Bi(NO_3_)_3_·5H_2_O, 1,3,5‐benzene tricarboxylic acid (H_3_BTC), and DCD as raw materials to synthesize Bi‐SAs/NC. NH_3_ released from DCD under high temperatures helped disperse Bi single atoms and dope N atoms into carbon networks. The whole synthesis process was recorded by in situ environmental transmission electron microscopy (ETEM) (Figure 5c). As‐prepared Bi‐SAs/NC performed FE(CO) of 97% at −0.5 V versus RHE.^[^ ^14b^ ^]^ Chen et al. chose UiO‐67 as the packaging material to encapsulate single Cu sites coordinated with the carbon atoms in the N‐heterocyclic carbene (HNC) molecule. The as‐obtained catalyst was denoted as 2Bn─Cu@UiO‐67, which performed a FE(CH_4_) of 81% at −1.5 V versus RHE, and the CH_4_ partial current density was up to −340.2 mA·cm^−2^. UiO‐67 was capable of stabilizing the HNC ligand, then the electron would transfer from the C atom in the HNC ligand to the single Cu site, which boosted the opportunity for the subsequent hydrogenation to produce CH_4_.^[^ ^128b^ ^]^ By extension, if researchers hoped to develop more MOF categories applied to SACs for eCO_2_RR, the following requirements could be met as far as possible: 1) Convenient synthesis; 2) Excellent conductivity; 3) the metal‐containing nodes of MOFs can have a certain performance on eCO_2_RR or the metal‐containing nodes can be easily replaced by other active metal sites; 4) Easy introduction of molecule catalyst (e.g., metal phthalocyanine) into MOF; 5) When the original metal‐containing nodes have to be eliminated through elevated temperature, acid etching, or other methods, the necessary metal active sites will not be eliminated simultaneously.

Apart from MOF‐derived SACs, other organic frameworks were also implemented to support single atoms. Covalent organic frameworks (COF), composed of organic building units through strong covalent bonds, are also platforms for SACs.^[^ ^129^ ^]^ Zhang et al. synthesized a conductive pyrazine‐linked 2D COF denoted as NiPc‐COF. It was speculated that NiPc‐COF presented a slipped AA (AA: one of the stacking models in COF) stacking structure and there appeared *π*–*π* stacking of 2D layers along the *c* direction. The distance of two adjacent Ni atoms was 22 Å, indicating the atomically dispersed active sites and the distance between the stacking 2D layers is 3.3 Å (Figure 5d). The in‐plane *π*–delocalization of monolayers and out‐of‐plane π–π stacking along the *c*‐axis efficiently boosted the conductive ability of NiPc‐COF. NiPc‐COF performed the maximum FE(CO) of 99.1% at −0.9 V versus RHE and the largest CO partial current density of −35 mA·cm^−2^ was obtained at −1.1 V versus RHE in an H‐cell (Figure 5e).^[^ ^41^ ^]^ The analogous work focusing on COF‐based catalysts for eCO_2_RR has also been studied by other researchers. Huang et al. incorporated CoPc into PDQ‐COF to form CoPc‐ PDQ‐COF. In an H‐cell with 0.5 m KHCO_3_, FE(CO) of CoPc‐ PDQ‐COF reached 96% at −0.66 V versus RHE with a current density of −49.4 mA·cm^−2^.^[^ ^129c^ ^]^ Cu─Tph‐COF‐Dct synthesized by Wang et al. realized an FE(CH_4_) of 80% and CH_4_ partial current density of −220.0 mA·cm^−2^ at −0.9 V versus RHE in a flow‐cell with 1.0 m KOH.^[^ ^130^ ^]^

As another class of COF, the covalent triazine framework (CTF) is also applied to eCO_2_RR.^[^ ^127^, ^131^ ^]^ Wu et al. changed the molar ratio of monomers 5,10,15,20‐tetrakis(4‐cyanophenyl)‐porphyrin (TPPCN) to 5,10,15,20‐tetrakis(4‐cyanophenyl)‐porphyrin‐Ni (Ni‐TPPCN), polymerizing them with sufficient ZnCl_2_ to fabricate different porous porphyrinic triazine frameworks PTF‐Zn, PTF‐ZnNi_x_ and PTF‐Ni_100_ (x referred to the molar percentage of TPPCN‐Ni). When the molar percentage of TPPCN‐Ni was too large, this spatial site separation strategy would be invalid, and the formation of Ni NPs remained inevitable. Powder X‐ray diffraction (PXRD) patterns confirmed that no identical peaks of Ni NPs were detected in Ni_5_‐PTF‐1000, while the corresponding peaks could be distinguished in Ni_20_‐PTF‐1000 and Ni_100_‐PTF‐1000. Ni_5_‐PTF‐1000 performed a FE(CO) of over 90% in the range from −0.6 to −1.0 V versus RHE, but Ni_20_‐PTF‐1000 and Ni_100_‐PTF‐1000 only performed a high FE(H_2_) of 48.2% and 81.5%, respectively, due to the gradually increasing content of Ni NPs.^[^ ^132^ ^]^ Up to now, there are still few applications of CTF for eCO_2_RR. While CTFs have tunable structures, abundant nitrogen sites, and high specific surface area, these features make it easier to capture metal atoms to construct isolated active sites. Meanwhile, avoiding the aggregation of isolated metal atoms in CTFs is also significant.^[^ ^133^ ^]^

Currently, to prepare SACs, researchers usually carbonize MOFs with metal atomic/ionic sites through high‐temperature pyrolysis and MOF‐based SACs will turn into carbon‐based SACs. There are still relatively few schemes to directly fix metal atom sites on MOFs/COFs/CTFs for eCO_2_RR.

### 3.3 Metal‐based and Oxide‐based SACs

When those single metal atoms are anchored on another metal material, the whole catalyst can be denoted as a single‐atom alloy (SAA). SAA has been a research frontier that is applied in various aspects due to its unique geometric and electronic structure. There exists a free‐atom‐liked state in the minority element of SAA, which endows SAA with the ability to alter adsorbate binding properties.^[^ ^134^ ^]^ Hung et al. realized the in situ formation of Fe single atoms on Cu to form Cu─Fe SAA with FE(CH_4_) of 64%. In situ Raman spectroscopy demonstrated that adsorption property and catalytic selectively changed due to the more intense peak of *CO_Fe_ than that of *CO_Cu_ so that *CO would attach to single Fe sites to undergo subsequent hydrogenation process.^[^ ^135^ ^]^

**Figure 6:**
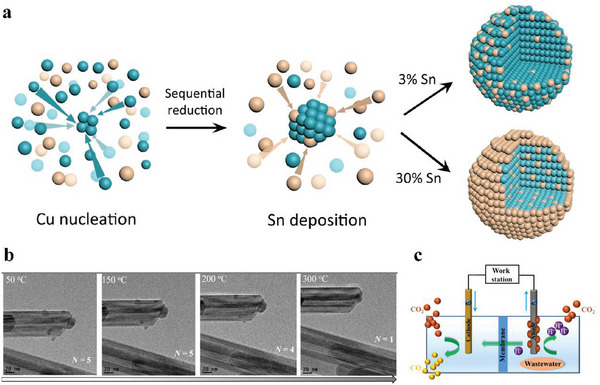
a) Schematic illustration of the Cu─Sn nanoparticle formation via sequential reduction.^[^ ^136a^ ^]^ Copyright 2021, Springer Nature. b) In situ ETEM acquired at different temperatures of Ag_NP_/MnO_2_.^[^ ^12b^ ^]^ Copyright 2020, American Chemical Society. c) Schematic diagram of a MEC‐CO_2_RR device.^[^ ^144^ ^]^ Copyright 2021, Elsevier.

In SAA, the interface structure between metal single atoms and metal atoms in supports works as the active sites for eCO_2_RR.^[^ ^45^, ^102^, ^136^ ^]^Zhang et al. synthesized a series of CuSn alloys with different feed molar ratios of Cu/Sn. With the increase of the feed molar ratio, the product selectivity would gradually shift from HCOOH to CO, performing a linear relationship between product distribution and CuSn alloy composition. When the feed molar ratio was up to 20, the Sn single atom doped Cu alloy, denoted as Cu_20_Sn_1_, reached a maximum FE(CO) of 95.3% at −1.0 V versus RHE. Compared with pure Cu foil, Cu_20_Sn_1_ SAA was able to reduce the formation energy of *COOH from 1.38 eV to 1.03 eV and the formation energy of CO from 1.27 eV to 0.56 eV. Meanwhile, competitive HER was impeded due to the synergistic effect of Cu and Sn atoms.^[^ ^137^ ^]^ Similarly, Ren et al. prepared CuSn alloy with different compositions through a sequential reduction process (**Figure** **6**a). The as‐prepared Cu_97_Sn_3_ SAA achieved the highest FE(CO) of 98% at −0.7 V versus RHE. Due to the lower formation energy of *CO than HCOOH and the increased energy of competitive HER, the Cu─Sn surface alloy such as Cu_97_Sn_3_ would tend to generate CO. Besides, the d‐band of Cu atoms in Cu─Sn alloy shifted closer to the Fermi level, which was in favor of CO_2_ adsorption.^[^ ^136a^ ^]^

Shen et al. investigated the dynamical evolution of Fe_1_‐Au interface sites. According to operando XAS characterization, with the decline of applied potential, the weaker Fe─O bond peak intensity and stronger Fe─Au bond peak intensity were observed. A reaction pathway was offered that the Au/Fe interface structure of O_3_‐Fe_1_Au_2_ would evolve into O_2_‐Fe_1_Au_3_ under working conditions. The low‐state Fe atom was eager to bond with the O atom in the CO_2_ molecule and the Au atom would bond with the C atom, thus this synergistic effect provided the ability to adsorb CO_2_ molecules and stabilize the *COOH intermediate.^[^ ^136b^ ^]^

Moreover, single atoms in SAA can also function as a modifier to help the host material undergo eCO_2_RR. Xie et al. synthesized Bi‐Pd SAA nano dendrites (ND) catalysts with different Bi/Pd ratios, and Bi_6_Pd_94_‐SAA ND catalyst demonstrated an optimal FE(CO) of 91.8% at −0.31 V versus RHE in a flow‐cell and 90.5% at −0.4 V versus RHE in H‐cell. The cyclic voltammetry (CV) measurement was conducted in CO_2_‐saturated 0.5 m KHCO_3_ to judge the ability of Bi‐Pd SAA ND catalysts to adsorb *H. The result suggested that Bi_6_Pd_94_‐SAA ND had the weakest *H adsorption peak and *H desorption peak, which can be the reason Bi_6_Pd_94_‐SAA ND was able to decline *H coverage on the surface to suppress H_2_, formate, and PdH production. XRD measurement confirmed that no Pd‐hydride was generated in the Bi_6_Pd_94_‐SAA ND catalyst after the stability test, further indicating the poor H* affinity of Bi_6_Pd_94_‐SAA ND catalyst after introducing single Bi atoms.^[^ ^138^ ^]^

Concerning oxide‐based SACs, Chen et al. and Wang et al. attempted to support single Cu atoms on Al_2_O_3_ and CeO_2_ respectively, and both of them realized the efficient conversion of CO_2_‐to‐CH_4_.^[^ ^48^, ^139^ ^]^ Zhang et al. successfully synthesized an Ag SAC. According to in situ ETEM and XRD, with the rising temperature, large‐size Ag nanoparticles collided strongly with the matrix, resulting in the surface reconstruction of MnO_2_, then the size of Ag nanoparticles would gradually shrink until it disappeared from the surface of the MnO_2_ matrix (Figure 6b) along with the preferentially exposed plane of MnO_2_ changing from (211) to (310). Then the Ag single atoms would be easy to be captured by oxygen atoms on the MnO_2_ (310) lattice plane to generate Ag_1_/MnO_2_, which showed an FE(CO) of over 90% from −0.7 V to −0.9 V versus RHE. This great performance could stem from H_2_O poisoning on MnO_2_ so that CO_2_ molecules processed more opportunities to be in touch with Ag single‐atom sites.^[^ ^12b^ ^]^ However, in the above work, there is no mention of the reconstitution of oxide substrates during eCO_2_RR. Ma et al. anchored atomically dispersed Cu atoms on Ag_2_S/Ag nanowires. After electrochemical treatment, S atoms in Ag_2_S/Ag nanowires were absent, companied with generated vacancies. This Cu SAC reached a FE(CO) ≈ 70% at −1.2 V versus RHE in an H‐cell with CO_2_‐saturated 0.1 m KHCO_3_.^[^ ^140^ ^]^ Similarly, this reconstruction of metal oxide substrates into metal monomers by electroreduction should exist, and this electrochemical treatment can be a method for the preparation of SAA. Up to now, in the field of eCO_2_RR, some researchers have studied the relationship between catalytic active sites and oxides and tried to synthesize more efficient catalysts of oxides.^[^ ^141^ ^]^ However, this part has not been systematically studied in the field of SACs.

In this Section, we classify and summarize several supports of SACs, including carbon materials, organic frameworks, metals, and oxides. Considering that the eCO_2_RR occurs in the aqueous phase and involves a large number of electron transfer processes, carbon materials have become the most promising supports. Carbon materials have good conductivity, a large specific surface area, and can adjust the morphology based on their precursors, which is conducive to the mass transfer of CO_2_ and electron transfer. Meanwhile, carbon materials have a certain degree of hydrophobicity, which can suppress the occurrence of competitive HER. Therefore, it is not difficult to find that in most of the examples mentioned above, high‐temperature carbonization steps are used for SACs supported on carbon substrates.

Organic framework materials, can anchor metal sites or adsorb metal ions during the synthesis process, and uniform carbon‐based catalysts can also be obtained after high‐temperature pyrolysis. However, there is still limited research on organic framework materials themselves as support. Attempting to take metal nodes in MOFs as reactive sites or to bind single‐atom sites internally for eCO_2_RR is a future research direction. As for COF and CTF materials, research methods focus on anchoring molecular catalysts to construct SACs. In the future, more SACs that can be applied to eCO_2_RR will be developed from COF and CTF themselves.

Single atoms on metal substrates also have excellent conductivity, but during the electron transfer process, due to the difference in the number of support metal atoms and atomically dispersed metal atoms, it is necessary to distinguish which part of the catalyst undergoes the reaction. Therefore, in metal‐based SACs, we may need to balance whether single‐atom sites exist as the main active sites or only as the assisted sites.

There is currently limited research on oxides‐based SACs applied to eCO_2_RR, and those oxides‐based SACs are usually used for thermal catalysis. In the field of eCO_2_RR, supports that have been applied include Al_2_O_3_, CeO_2_, and MnO_2_. Oxide supports can effectively anchor single‐atom sites to construct SACs, but their inherent reaction inertness and weak conductivity make them not very suitable for electrocatalytic processes.

## 4 eCO_2_RR Toward Valuable Products

Currently, the products of eCO_2_RR on SACs are CO or formate, accompanied by a small part of CH_3_OH, CH_4,_ and other C_2+_ products. Various products point to varied electron transfer numbers, different reaction pathways, or different catalytic active sites. In this section, we will introduce the application of SACs in obtaining diverse products based on the classification of products and suggest strategies and means to improve product selectivity.

### 4.1 CO_2_ to CO

Gaseous CO is one of the simplest products during the process of eCO_2_RR. As mentioned above, the conversion of CO_2_‐to‐CO undergoes a two‐electron transfer: 1) CO_2_ + e^−^ + H^+^ → *COOH; 2) *COOH + e^−^ + H^+^ → *CO + H_2_O; 3) *CO → CO. Those common Fe, Co, Ni SACs merely produce CO in the period of eCO_2_RR. As mentioned above, to avoid metal agglomeration during synthesis, the number of active sites in SACs usually is far lower than those in metal nanoparticle catalysts or metal bulk catalysts. Afterward, several potential problems were exposed: 1) lack of ability to break linear scaling relationship; 2) difficulty in capturing CO_2_ molecules in the traditional aqueous electrolyte; 3) lower current density. Based on numerous studies on the conversion of CO_2_‐to‐CO on SACs,^[^ ^142^ ^]^ researchers are not satisfied with these statuses, and more efficient methods to produce CO are still proposed, including tandem strategy,^[^ ^143^ ^]^ bio‐electrochemical system,^[^ ^144^ ^]^ ionic liquid (IL) electrolyte,^[^ ^145^ ^]^ current density enhancement strategy.^[^ ^36^, ^146^ ^]^

SACs enable participation in the construction of a tandem system. A Tandem catalyst can combine two catalysts and integrate their advantages, while their disadvantages will be evaded. CoPc©Fe─N─C synthesized by Lin et al. successfully promoted CO desorption on CoPc and inhibited competitive HER on Fe─N─C, obtaining FE(CO) over 90% at a wide range of −0.13 to −0.84 V versus RHE with a durable and steady current density.^[^ ^143a^ ^]^ Chen et al. developed a Cu‐based tandem catalyst denoted as Cu─S_1_N_3/_Cu_x_ with FE(CO) of 100% at −0.65 V versus RHE. The N, S co‐coordinated Cu sites optimized the binding energy and strength of the intermediates. The existence of adjacent Cu_x_ clusters not only aided the N, S co‐coordinate Cu sites to decrease the formation energy of *COOH intermediate but also gave rise to water dissociation, leading to a fastened protonation process of adsorbed CO_2_^−^.^[^ ^143b^ ^]^

The combination of bio‐electrochemical systems and SACs provides more ideas to deal with CO_2_ emitted from bacteria. Li et al. designed a bio‐electrochemical system containing Fe SA‐NC cathode and bioanode. First, the microorganisms oxidized the organics at the bioanode. Electrons were generated and then transferred to the cathode to help drive CO_2_RR with assistant voltage. The microbial electrolysis cell (MEC) (Figure 6c) equipped with cathodic Fe SA‐NC (MEC_Fe SA‐NC_) needed a lower input voltage than MEC_NC_ at varied currents from 0.5 to 2.0 mA because Fe SA‐NC had a lower overpotential of CO_2_RR. At the constant current of 1.5 mA, MEC_Fe SA‐NC_ performed a CO production rate of 33.66 ± 0.58 mmol g^−1^_cat_·h^−1^, which was 4‐fold higher than that of MEC_NC_.^[^ ^144^ ^]^

ILs, merely composed of cations and anions, are organic salts that keep a liquid state below 100 °C.^[^ ^147^ ^]^ ILs with product selectivity enable more CO_2_ dissolution than conventional aqueous electrolytes, which are also considered to reduce the energy potential barrier of CO_2_RR and make the reaction proceed at a low overpotential.^[^ ^148^ ^]^ Ren et al. impregnated [BMIM][PF_6_] ILs into the channels and pores of a Ni─N catalyst. Ni─N@ILs displayed a significantly improved CO partial current density of −66.1 mA·cm^−2^ at −1.0 V versus RHE and that of catalysts without impregnating into ILs was only ≈ 40% at the same applied potential. Meanwhile, Ni─N@ILs also obtained a maximum FE(CO) of 98% at −0.7 V versus RHE. Under the reaction condition incorporating ILs, a solid‐liquid interface with high CO_2_ concentration was formed and beneficial for eCO_2_RR.^[^ ^145a^ ^]^ A Mn SAC, Mn─C_3_N_4_/CNT, with unique Mn─N_3_ sites was fabricated by Feng and co‐workers. In a CO_2_‐saturated 0.5 m KHCO_3_ electrolyte, the highest CO partial current density of −22.4 mA·cm^−2^ was obtained at −0.75 V versus RHE. As a comparison that the electrochemical measurement conducted in a CO_2_‐saturated IL electrolyte ([Bmim]BF_4_)/acetonitrile (CH_3_CN)‐H_2_O), a higher CO partial current density of −29.7 mA·cm^−2^ could be achieved at overpotentials of 0.62 V.^[^ ^145b^ ^]^

Due to the low loading of center metal atoms, it is quite difficult for SACs to achieve a high current density that is unable to reach nearly an industry level (over −100 mA·cm^−2^). Yang et al. produced a high‐yield, flexible, and self‐supported Ni SAC denoted as NiSA/PCFM, which was applied in flow‐cell and performed an outstanding CO partial current density of −336.5 mA·cm^−2^ at −1.2 V versus RHE, and FE(CO) reached 83% at the same potential. Furthermore, NiSA/PCFM even maintained the stability of 120 h with a decreasing FE(CO) of <5% of the initial value. This high current density and long‐term stability originated from no polymer binders to connect catalysts in the gas diffusion layer.^[^ ^146b^ ^]^ Liu et al. introduced lanthanoid Gd atoms into the normal Ni catalyst, gaining a Gb/Ni co‐doped catalyst CBNNiGb‐700. Ni species would generate Ni nanoparticles encapsulated in the carbon layer and Ni single‐atom sites supported on the carbon surface. The large atom radius of Gb aroused defects generation during the agglomeration of Ni atoms so that the size of Ni nanoparticles, as well as HER activity on the Ni nanoparticles, was suppressed. Meanwhile, according to the XPS Ni 2p spectrum and Gb 4d spectrum, electrons in the Ni *3d* orbitals were pulled to higher energy levels due to the strong lanthanide contraction effect of Gb atoms, successfully improving the catalytic activity and strengthening the *COOH intermediate adsorption. Finally, CBNNiGb‐700 kept the high current density from Ni nanoparticles and high selectivity from single Ni atom sites simultaneously. In a flow‐cell, CBNNiGb‐700 demonstrated a current density of −308 mA·cm^−2^ with a high FE(CO) of 97% at −0.91 V versus RHE, approaching the industrial demand.^[^ ^36^ ^]^

Sometimes, the original material can be modified by additional single‐atom sites and they can also be the accessory sites for eCO_2_RR. Ni et al. took Fe‐containing and nitrogen‐rich g‐C_3_N_4_ as the precursor to get Fe SACs (DNG‐SAFe, DNG: graphene‐like porous carbon with rich intrinsic defects and doping N atoms) with abundant intrinsic defects and Fe─N_4_ sites. NG‐SAFe (NG: graphene‐like porous carbon without intrinsic defects) and DNG without Fe─N_4_ sites as control samples were also prepared. Among these samples, DNG‐SAFe performed the best FE(CO) of 90% at −0.75 and −0.85 V versus RHE in an H‐cell with CO_2_‐saturated 0.1 m KHCO_3_. Subsequently, DNG‐SAFe with SCN^−^ poisoning performed similar LSV curves and FE(CO) to those of DNG‐SAFe without SCN^−^ poisoning, while the performance of NG‐SAFe was dramatically inhibited, indicating that intrinsic defects were the main active sites but not Fe─N_4_ sites. DNG‐SAFe also had a larger ESCA‐normalized j_CO_ than DNG, thus the synergistic effect between intrinsic defects and Fe─N_4_ sites could promote the catalytic activity of DNG‐SAFe.^[^ ^149^ ^]^ Zhang et al. supported Sn single‐atoms on Cu_2_O nanosheets to form the structure of Sn─O─Cu, which kept Cu atoms with the valance of +1. The in situ Cu K‐edge spectra of Cu_2_O and Sn/Cu_2_O demonstrated that the XANES spectral feature of Sn/Cu_2_O wouldn't change when the applied potential was below −0.4 V versus RHE, whereas the XANES spectral feature of Cu_2_O was similar to that of metallic Cu at −0.4 V versus RHE, manifesting Cu^+^ of Sn/Cu_2_O was more stable. With the coordination of Sn single atoms, FE(CO) of Sn/Cu_2_O reached ≈ 85%, while FE(H_2_) decreased to 15%.^[^ ^150^ ^]^ Peng et al. even stabilized K single atoms through Cu─F bond and K─F bond in KCuF_3_ during the electrochemical reduction process. K single atoms inhibited the break of C─O bond and promoted subsequent intermediate hydrogenation to C_2_H_5_OH.^[^ ^151^ ^]^

Based on the former strategies for improving eCO_2_RR, the conversion of CO_2_‐to‐CO on SACs is much more mature than other products. However, another important problem for this reaction is how to effectively separate reactants and products. Besides, considering the highly dispersed active sites in SACs, the products during eCO_2_RR focused on CO and it was rare for them to reduce CO_2_ more deeply to products such as CH_4_ or even C_2+_ products. Hence, adopting the unique electronic structure of single atoms to modify the conventional materials to realize high performance or obtain other products is also a practicable method.

### 4.2 CO_2_ to Formate

HCOOH is one of the common liquid products in eCO_2_RR, which can be obtained through a 2‐electron pathway.^[^ ^152^ ^]^ Different from the key intermediate *COOH of CO, the first electron‐proton step in the conversion of CO_2_‐to‐HCOO^−^ mainly generates *OCOH, and then the second electron‐proton transfer step is to form HCOO^−^ or HCOOH.

In recent years, several metal atoms, such as Mo, Bi, and Sb, have been used as the center sites of SACs to produce formate from CO_2_.^[^ ^13^, ^43^ ^]^ Shang et al. designed a single‐atom In^δ+^‐N_4_ interface with a FE(HCOOH) of 96% at −0.65 V versus RHE and a maximum TOF of 12500 h^−1^ at −0.95 V versus RHE. In situ XAFS characterization verified when the catalyst was immersed in CO_2_‐saturated 0.5 m KHCO_3_, the oxidation state of the center In atom would rise. The bond of In─N would be shortened from 1.95 to 1.93 Å at −0.65 V versus RHE, indicating the robust catalytic activity of the catalyst.^[^ ^13a^ ^]^ Zu et al. supported kilogram‐scale atomically dispersed Sn^δ+^ on N‐doping graphene through a quick freeze‐vacuum drying‐calcination method, reaching an FE(HCOOH) of 74.3% at‐1.6 V versus SCE. In situ FTIR was employed to disclose the onset potential of the catalyst. The spectra manifested that the characteristic peak of asymmetric O─C─O stretches assigned to HCOO^−^_ad_ suddenly appeared at −0.74 V versus SCE, corresponding to an overpotential of only 60 mV. Low onset potential might originate from the exergonic formation energy of CO_2_^•−*^ (intermediate formed by CO_2_ activation) and HCOO^−*^, hence the process of CO_2_ activation and protonation will occur simultaneously.^[^ ^13c^ ^]^

Except for those common SACs with main group metal atoms, Xie et al. developed a NiSn atomic pair on an integrated electrode. NiSn‐APC afforded formate productivity of 36.7 mol h^−1^·g_Sn_^−1^ and a TOF of 4752 h^−1^. The existence of the adjacent Ni atom was advantageous for decreasing the barrier of converting CO_2_ to *OCOH from 0.78 to −0.05 eV on the Sn atom, whereas the conversion of CO_2_‐to‐*COOH was thermodynamically unfavorable, hindering CO production.^[^ ^44^ ^]^

To avoid blending HCOOH with electrolyte, Zheng et al supported Pb single atoms on Cu host material to generate Pb_1_Cu SAA catalyst, reaching a FE(HCOOH) of 96% at −0.80 V versus RHE with a partial current density of −800 mA·cm^−2^ and longtime stability of 180 h. The electrochemical measurement was conducted in a 3‐cm^2^ electrode device with a proton‐conducting solid electrolyte. Under the effect of the electric field, HCOO^−^ could be easily transferred into the middle solid‐electrolyte channel and then react with H^+^ at the anode side to generate HCOOH. Eventually, the mixture of HCOOH and H_2_O was obtained. According to ATR‐FTIR and DFT calculations, it was estimated that formate intermediates were probably adsorbed on Cu atoms and doped Pb atoms had an impact on tunning the geometric and electronic structures of the whole catalyst to promote selectivity and activity for formate.^[^ ^153^ ^]^

The catalysts aimed at HCOO^−^ or HCOOH during eCO_2_RR are relatively simple, and mainly dominated by those main family elements (e.g., Sb, Sn, In). The content of HCOOH is usually measured by ^1^H NMR spectroscopy, while it is difficult to separate HCOO^−^ or HCOOH from electrolyte after reaction, the practical application of electrochemical CO_2_‐to‐HCOOH is still limited. In the future, researchers should pay more attention to the separation of reactants and products.

### 4.3 CO_2_ to CH_4_

**Figure 7:**
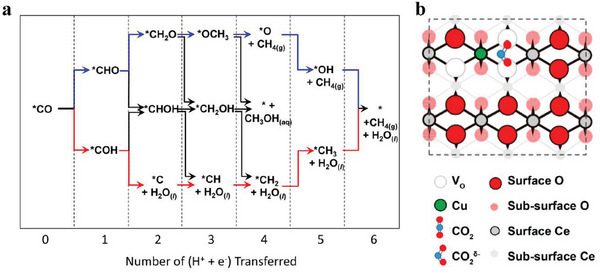
a) Schematic description of reduction pathways for the conversion of *CO‐to‐CH_4_/CH_3_OH.^[^ ^157^ ^]^ Copyright 2016, American Chemical Society. b) The most stable structure of Cu‐doped CeO_2_(110) with three O vacancies, on which CO_2_ is activated.^[^ ^139^ ^]^ Copyright 2018, American Chemical Society.

As an industrial feedstock and a common energetic fuel, CH_4_ can be directly used as a substitute for gasoline or further converted to CO and H_2_. Consequently, the conversion of CO_2_‐to‐CH_4_ may play an important role in future industrial systems.^[^ ^154^ ^]^ However, as the deepest C_1_ product during eCO_2_RR, conversion of CO_2_‐to‐CH_4_ requires undergoing an eight‐electron pathway and forming seven intermediates.^[^ ^155^ ^]^ It is generally considered that hydrogenation of *CO is the rate‐determining step of the conversion of CO_2_‐to‐CH_4_, hence the formation of CH_4_ has to suppress *CO desorption and C─C coupling simultaneously.^[^ ^155^, ^156^ ^]^ Moreover, the hydrogenation of *CO can obtain two types of intermediates of *CHO and *COH (**Figure** **7**a).^[^ ^157^ ^]^ Thus it can be seen, that how to obtain *H from H_2_O in the process of conversion of CO_2_‐to‐CH_4_ is also a complex problem. As for homogenous active sites in SACs, it is difficult for them to realize simultaneously efficient electroreduction of CO_2_ and H_2_O molecules. Hence, this complicated reaction pathway makes the conversion of CO_2_‐to‐CH_4_ hard to realize on SACs.

In terms of traditional carbon‐based SACs, Han et al. attempted to anchor Zn single atoms on microporous N‐doped carbon to generate SA‐Zn/MNC, reaching a maximum FE(CH_4_) of 85% at −1.8 V versus SCE with the partial current density of −31.8 mA·cm^−2^. DFT calculations manifested that *OCHO was the firstly generated intermediate, and H atoms in *OCHO were eager to bond to C atoms around Zn single atom sites to help stabilize the intermediate, leading to a lower formation energy (0.46 eV) than *COOH intermediate (1.2 eV).^[^ ^49^ ^]^ Cai et al. adopted a semi‐transformed strategy to calcine Cu‐doped metal‐organic complex precursor at relatively low temperatures to make Cu single atoms coordinated with O and C atoms simultaneously. Based on in situ UV–vis absorption spectra, no change was found in as‐prepared Cu‐CD (Cu supported on carbon dots) during electrolysis, indicating that Cu single‐atom sites were quite stable and worked as the intrinsic active sites. Moreover, a high *H adsorption energy suppressed the competitive HER, and the lowest U_L_ (limiting potential) of CH_4_ led to a high selectivity toward CH_4_ and a maximum FE(CH_4_) of 78% at −1.44 V versus RHE.^[^ ^50^ ^]^ It was noteworthy that Guan et al. prepared a series of Cu, N‐doped carbon nanosheet catalysts with various Cu loading and coordination environments. A lower Cu loading amount (2.4% mol) made the active sites exist as isolated Cu─N_2_ and Cu─N_4_ species, which impeded the C─C coupling process, and the product was mainly CH_4_ with FE(CH_4_) of 38.6% at −1.6 V versus RHE.^[^ ^51^ ^]^

The conversion of CO_2_‐to‐CH_4_ is more difficult on traditional carbon‐based SACs than that of CO_2_‐to‐CO, hence some researchers started to introduce a synergetic effect between single active sites and support or develop a tandem strategy to enhance this process. Chen et al. synthesized two kinds of Cu SACs using Al_2_O_3_ and Cr_2_O_3_ as substrates. Due to the stronger Lewis acidity of Al_2_O_3_, Cu/C‐Al_2_O_3_ processed a better performance in converting CO_2_ to CH_4_ with the highest FE(CH_4_) of 62% at −1.2 V versus RHE and a current density of −153.0 mA·cm^−2^. DFT calculations explained that *CH_4_O had lower formation energy than competitive CH_3_OH, hence CH_4_ was the main product instead of CH_3_OH.^[^ ^48^ ^]^ Wang et al. introduced three O vacancies around each Cu single‐atom site in Cu‐doped CeO_2_ nanorods. The strong activation from Cu single‐atom sites and the surrounding three O vacancies made the adsorbed CO_2_ molecule be in the form of bench‐structure, promising CO_2_ could be reduced to value‐added products (Figure 7b). Cu─CeO_2_‐4% performed a peak FE(CH_4_) of 58% and kept the FE(CH_4_) over 40% in 8000 s electrolysis at −1.8 V versus RHE. Due to the oxophilicity of the Cu‐substituted CeO_2_ and the highly dispersed Cu sites, the pathway to CH_3_OH or other C_2+_ products was impeded.^[^ ^139^ ^]^ Recently, Cu_1_‐CeO_2_ synthesized by Jiang and co‐workers also reached an FE(CH_4_) of 67%. Through XANES spectra, they discovered that the formation of Cu─O─Ce bond maintained the high valance of Cu^2+^ single‐atom site, which preferred *CO hydrogenation to C─C coupling during eCO_2_RR.^[^ ^158^ ^]^ Jiao et al. proposed a molecular scaffold strategy. According to DFT calculation, the molecular scaffold of g‐C_3_N_4_ and N‐doped graphene could be used as an additional active site, thus constructing a dual active site with a synergistic effect. Taking CH_4_ production as an example, the C atoms of the intermediates (e.g., *COOH, *CO, *CHO) during the first half of the reactions would form a strong bond with the Cu atoms. The oxygen atoms in the intermediates (e.g., *OCH_2_, *O, *OH) during the latter half of the reaction tended to bind with the carbon atoms in the scaffold. Experiments also confirmed that molecular scaffolded Cu─C_3_N_4_ had higher FE(CH_4_) or FE(CH_3_OH) than Cu─NC (single Cu supported on g‐C_3_N_4_ and N‐doped graphene, respectively). Due to the unsymmetric characteristics, Cu─C_3_N_4_ could also produce C_2_ species, providing a new idea for achieving deeply reduced products such as C_2_H_5_OH, C_2_H_4_, or C_2_H_6_ in eCO_2_RR.^[^ ^159^ ^]^

In the above reports, Lewis acidity and oxophilicity were mentioned when they were chosen as supports to load single‐atom sites. Those metal oxides with strong Lewis acidity can promote metal atoms to interact with the O atoms in CO_2_ molecules through Lewis acid‐base interactions, facilitating the cleavage of C─O bonds. The presence of Lewis acid sites can modulate the electronic structure of the single‐atom sites and stabilize the intermediates in eCO_2_RR.^[^ ^48^ ^]^ Supports with strong Lewis acidity are often used in CO_2_ methanation.^[^ ^160^ ^]^ On the support with better oxophilicity, the O atom in the *CHO or *COH would be more likely to be fixed on the support than remain in the intermediates to form CH_3_OH.^[^ ^139^, ^161^ ^]^ Similarly, this situation may exist in the selective production of C_2_H_4_ or C_2_H_5_OH during eCO_2_RR. Thus, the support effect can also play a key role in SAC design to alleviate the problem that homogenous active sites are unable to produce value‐added products than CO or formate.

Taking advantage of tandem catalysts, the conversion of CO_2_‐to‐CH_4_ can be divided into two steps, which efficiently avoids the problem that it is difficult for CO_2_ on SACs to undergo an eight‐electron transfer. Lin et al. ultrasonically mixed Zn─N─C and CoPc to establish a tandem catalyst. CO_2_ was first reduced to CO on CoPc, then CO was desorbed from CoPc and re‐adsorbed on Zn sites in Zn─N─C. Since HER easily occurs on pyridine nitrogen of the ZnN_4_ site, *CO and *H co‐existed on the ZnN_4_ site, hence the key intermediate *CHO to CH_4_ evolution was conveniently generated. The resulting CH_4_/CO producing rate was 100 times enhanced over individual CoPc or Zn─N─C catalysts.^[^ ^162^ ^]^ This strategy can be more commonly used in generating C_2+_ products, which will be discussed in the later part.

### 4.4 CO_2_ to CH_3_OH

CH_3_OH is another expected C_1_ product from eCO_2_RR, whose conversion follows a six‐electron pathway. The conversion of CO_2_‐to‐CH_3_OH is competitive with the conversion of CO_2_‐to‐CH_4_ (Figure 7a).^[^ ^163^ ^]^ After one hydrogenation step and three electron‐proton transfer steps, in which *CHO, *CH_2_O, *CH_3_O, and *CH_4_O intermediates are generated in sequence, the oxophilic intensity of the active site will decide that *CH_4_O intermediate will desorb as the form of either CH_3_OH or CH_4_.^[^ ^163^, ^164^ ^]^ The post‐processed products of CH_3_OH, such as dimethyl ether, C_2_H_4_, and beyond, are also important industrial feedstocks.^[^ ^165^ ^]^ To date, a lot of electrode materials have been developed to convert CO_2_ into CH_3_OH, while little attention focuses on SACs.^[^ ^166^ ^]^

Wu et al. discovered that the conversion of CO_2_‐to‐CH_3_OH was a domino process, in which CO_2_ was first reduced to CO after a two‐electron transfer and then underwent a four‐electron transfer to produce CH_3_OH. FePc, CoPc, and NiPc were anchored on a highly conductive carbon network, respectively. FePc/CNT and NiPc/CNT only produced CO and H_2_ at a wide range of applied potentials, while CH_3_OH was only detected on CoPc/CNT with the onset potential of −0.82 V versus RHE. The maximum FE(CH_3_OH) of 44% and the largest partial current density of −10.6 mA·cm^−2^ were achieved at −0.94 V versus RHE in a flow‐cell with 0.1 m KHCO_3_.^[^ ^32a^ ^]^

To maximize the catalytic activity, Yang et al. adopted an electrospinning method to embed pre‐synthesized Cu/ZIF‐8 nanoparticles into polyacrylonitrile (PAN) nanofibers. Cu single atoms doped carbon nanofibers with through holes (CuSAs/TCNFs) were obtained via carbonization and acid etching. Moreover, CuSAs/TCNFs had higher ECSA (23.3 mF·cm^−2^) than CuSAs/CNFs (7.2 mF·cm^−2^), indicating the existence of the through‐hole structure successfully diffused Cu single‐atom active sites into the whole catalyst. CuSAs/TCNFs performed a maximum FE(CH_3_OH) of 44% along with FE(CO) of 56% at −0.9 V versus RHE. In the light of DFT calculations, the conversion of CO_2_‐to‐*COOH was a rate‐determining step, and the Cu─N_4_ sites in CuSAs/TCNFs exhibited higher free energy (1.17 eV) than Ni─N_4_ sites (0.98 eV). Meanwhile, the desorption energy of *CO intermediate on Cu─N_4_ sites was a slightly thermodynamical uphill process (0.12 eV), hence *CO intermediate would undergo subsequent reactions. However, due to the loss of C─C coupling and the high formation energy of the key *C intermediate to CH_4_, the final products were limited to CH_3_OH and CO.^[^ ^46^ ^]^

Zhao et al. prepared ultrathin layers which immobilized Cu single atoms (SA─Cu─MXene), performing a high FE(CH_3_OH) of 59.1% at −1.4 V versus RHE. SA─Cu─MXene was synthesized by selective etching the quaternary MAX phase containing both Cu atoms and Al atoms. Al atoms would react with molten ZnCl_2_ at 600 °C to form AlCl_3_, which was easy to sublimate at that temperature, hence Al atoms were selectively etched, and Cu atoms were left alone so that accordion‐like MXene was generated. To further obtain SA─Cu─MXene, sonication was applied to exfoliate the MXene. Except for high selectivity for CH_3_OH, SA─Cu─MXene presented longtime stability of 30 h with FE(CH_3_OH) over 58% simultaneously. However, its working potential was more negative than other catalysts, due to its poor conductivity. Based on DFT calculations, the rate‐determining step was the conversion of HCOOH*‐to‐CHO*. In addition, the energy consumption of the rate‐determining step on SA─Cu─MXene was 0.38 eV lower than on Cu‐particles‐MXene, indicating SA─Cu─MXene was more in favor of generating CH_3_OH.^[^ ^47^ ^]^

In the selective competition between CH_4_ and CH_3_OH, the presence of O atoms will affect the selectivity of the products and it is a feasible solution to try to regulate the oxophilic intensity of supports or active sites to control the retention and removal of oxygen atoms in products. Meanwhile, different from CO or formate, CH_4_ and CH_3_OH are usually generated on Cu‐based catalysts in that Cu atoms have a moderate binding affinity of *CO, promoting subsequent hydrogenation step and hindering the desorption of *CO intermediate or high attractiveness for *H, which originate from too weak or too strong *CO binding affinity.^[^ ^167^ ^]^ However, this situation can lead to a wide variety of products during eCO_2_RR (C_1_, C_2+_, and H_2_). So how researchers can ensure high selectivity of CH_3_OH and CH_4_ on SACs remains a challenging task.

### 4.5 CO_2_ to C_2+_ Products

C_2+_ products (C_2_H_4_, C_2_H_5_OH, etc.) have higher energy density and value compared with C_1_ products, so scientists have begun to look at obtaining these products through CO_2_RR.^[^ ^168^ ^]^ Compared to those C_1_ products, the formation of C_2+_ products can be rougher in that only one CO_2_ molecule should participate in the reaction to form C_1_ products, while two CO_2_ molecules are necessary during the formation of C_2+_ products. When CO_2_ molecules are captured by the catalytic active sites and reduced to *CO, two adjacent *CO intermediates undergo C─C coupling to form OC─CO or OC─CHO intermediate, which then selectively yields C_2_H_4_ or C_2_H_5_OH through subsequent hydrogenation and dehydration processes.^[^ ^169^ ^]^ To promote the C─C coupling process, two reactive active sites, separately containing *CO or *COH intermediates, usually are expected to get as appropriately close to each other. This harsh condition makes it difficult for SACs to generate C_2+_ products because single‐atom active sites that are too close tend to aggregate. In addition, Cu‐based catalysts are the main classification of electrocatalysts that can achieve this reaction and are simultaneously faced with other complex problems at present. Generally, achieving the conversion from CO_2_ to C_2+_ products on a single component Cu‐based electrocatalyst involves not only the continuous transfer steps of more than ten electron‐proton pairs but also the C─C coupling step between two adjacent *CO intermediates. Sometimes, the key intermediate *CO tends to be directly desorbed to generate CO on most electrocatalysts, which makes the one‐step conversion process from CO_2_ to C_2+_ products particularly difficult with the low product selectivity. Therefore, the reaction on single‐component Cu‐based electrocatalysts to obtain C_2+_ products is facing the problems of high overpotential and low Faraday efficiency.

In the case of SACs, due to the conflict between the low metal loading of SACs and the longer distance of adjacent metal sites for C─C coupling, it seems much more difficult for highly dispersed single‐atom sites in SACs to obtain C_2+_ products.^[^ ^51^, ^170^ ^]^ Meanwhile, the catalytic mechanism for generating C_2+_ products is still controversial.^[^ ^4^ ^]^

#### 4.5.1 Ethanol

**Figure 8:**
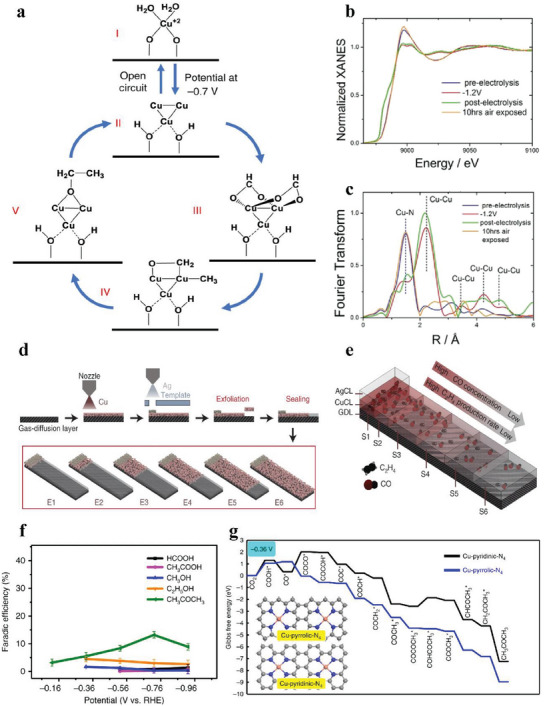
a) The hypothesized reaction mechanism to produce C_2_H_5_OH on Cu SA.^[^ ^35b^ ^]^ Copyright 2019, Springer Nature. b) Comparison between the K‐edge XANES experimental spectra and c) Fourier transform of the experimental EXAFS spectra of Cu_0.5_NC under no potential applied (blue line), Cu_0.5_NC during electrolysis at −1.2 V versus RHE (red line), after electrolysis under no potential applied (green line), Cu_0.5_NC after electrolysis at −1.2 V versus RHE and then exposed to air (orange line).^[^ ^35a^ ^]^ Copyright 2019, Wiley‐VCH. d) Schematic of the preparation procedure of s‐GDE. The geometries of six s‐GDEs (from E1 to E6) with a constant dimension of the Ag CL (L: 0.20 cm, W: 0.50 cm) and a varied dimension of the Cu CL (L: 0.20 – 2.00 cm, W: 0.50 cm) are shown in the inset; e) Schematic of decreasing C_2+_ mass activity, along with the decreasing CO concentration along the y axis of s‐GDE.^[^ ^174^ ^]^ Copyright 2022, Springer Nature. f) Faradaic efficiency of CO_2_ reduction products on Cu‐SA/NPC_Ar_; g) Free energy diagrams calculated at a potential of −0.36 V versus RHE for CO_2_ reduction to CH_3_COCH_3_ on Cu‐pyridinic‐N_4_ and Cu‐pyrrolic‐N_4_ sites of Cu‐SA/NPC (the computational models are included in the figure).^[^ ^35c^ ^]^ Copyright 2020, Springer Nature.

Xu et al. discovered the formation of Cu clusters in Cu SAC during the conversion of CO_2_‐to‐ CH_5_OH, obtaining an FE(CH_3_CH_2_OH) of 91% at −0.7 V versus RHE. The operando XAS result disclosed that the atomically dispersed Cu^2+^ species in the catalyst would be transformed into metallic Cu_3_ or Cu_4_, which become the real active sites to promote the conversion of CO_2_‐to‐CH_3_CH_2_OH. The hypothesized reaction mechanism suggested that an electron would transfer from the carbon substrate to Cu^2+^, and Cu^2+^ would be reduced to Cu^0^. Then those close Cu^0^ would aggregate into Cu_3_ or Cu_4_. Cu clusters would link with the surface hydroxyl group and bind to CO_2_ in the electrolyte as a transient active site, and then complete the reaction through continuous steps of the proton‐electron transfer. Without applied voltage, Cu_3_ or Cu_4_ was highly unstable and would be easily oxidized by weak oxidants such as dissolved CO_2_ and then reduced back to Cu single atom to complete the catalytic cycle, hence the Cu clusters worked as the real active sites in practice (**Figure** **8**a).^[^ ^35b^ ^]^

A similar dynamical evolution process was also observed by Karapinar and co‐workers. To reach the optimal result, the electrochemical measurement of Cu_0.5_NC was conducted in a flow‐cell with CO_2_‐saturated 0.1 m CsHCO_3_ under a closed CO_2_ volume of 300 mL cycled through the electrolyze at a flow of 2.5 mL min^−1^. Therefore, a maximum FE(CH_3_CH_2_OH) of 55% was attained at −1.2 V versus RHE. The oxidation state of Cu atoms decreased from +2 to 0 and Cu─Cu bindings were formed during the electrolysis according to operando XAS characterization, while no metallic copper phase and the restoration of the original spectrum after exposing the material to air (Figure 8b‐c).^[^ ^35a^ ^]^ It seems that the dynamical evolution of dispersed Cu singe atom sites to Cu clusters can promote C─C coupling and subsequent C_2+_ products. Meanwhile, the researchers discovered that the size of the cation in the electrolyte would influence the result a lot. The FE(CH_3_CH_2_OH) would be promoted by increasing the cation size (Li^+^< Na^+^< K^+^< Cs^+^) in the electrolyte in that the stronger cation hydration of larger cation size reduced the cation‐specific adsorption on the cathode and limited HER.^[^ ^35a^ ^]^

Lakshmanan et al. introduced carboxyl groups, Nafion coating, and Fe single‐atom sites on multi‐walled carbon nanotubes through modification measures such as concentrated nitric acid treatment, solution casting, and ion exchange, respectively. Due to the electrostatic interaction between Fe single‐atom sites and carboxyl groups on carbon nanotubes, the original Fe─(O)_3_ conformation was deformed during CO_2_RR and stabilized by carboxyl functional groups, which made the valence state of Fe single‐atoms stable near +3 and ensured excellent CO_2_ reduction to CO. The generated CO was transferred to the functionalized multi‐walled carbon nanotubes for further reduction to CH_3_CH_2_OH with an FE over 40%.^[^ ^171^ ^]^

#### 4.5.2 Ethylene

Kusama et al. immobilized the crystallized Cu phthalocyanine (CuPc) on carbon black, achieving a FE(C_2_H_4_) of 25% at −1.6 V versus Ag/AgCl in an H‐cell with CO_2_‐saturated 0.5 m KCl, while the control group of non‐crystallized CuPc displayed no selectivity to C_2_H_4_. The researcher concluded that the crystallinity of CuPc might have an impact on the selectivity of C_2_H_4_.^[^ ^170^ ^]^ Ma et al. prepared confined copper catalysts by anchoring Cu atoms on a CTF, featuring its initial CuN_2_Cl_2_ structure. CTF‐Cu‐4.8% performed a maximum FE(C_2_H_4_) of 30.6% at −1.47 V versus SHE in an H‐cell. Operando XAFS analysis revealed the dynamic formation of copper atom clusters, confirming that in situ‐formed copper atom clusters were the real active sites of CO_2_RR.^[^ ^172^ ^]^

As the key intermediate, *CO plays an important role in eCO_2_RR, and increasing the coverage of *CO might be a promising way to improve the performance of producing C_2+_, which was confirmed by the following research.^[^ ^173^ ^]^ Meng et al. designed a tandem catalyst that connected porphyrinic triazine frameworks anchored with atomically dispersed nitrogen‐nickel sites PTF(Ni) to Cu clusters. During the reaction process, the Ni single atom in PTF(Ni) molecule could efficiently reduce CO_2_ to CO, then CO would diffuse to nearby Cu clusters, forming a high CO coverage which was beneficial for producing C_2_H_4_. The resulting FE(C_2_H_4_) reached 57.1% with a partial current density of −3.1 mA·cm^−2^.^[^ ^35d^ ^]^ Zhang et al. designed stacked segmented gas diffusion electrodes (s‐GDE) composed of Ag and Cu catalyst layers (CL). A condensed 0.2 cm long Ag CL for generating CO was stacked on top of a Cu CL whose length was regulated from 0.2 to 2.0 cm. The longer Cu CL would prolong the residence time of CO on Cu CL to achieve better performance for C_2+_ products (Figure 8d,e). As the part to produce CO, Ag CL must be placed at the inlet to take advantage of the current along the channel gradient and enhance the coverage of *CO. When the Ag CL was replaced by Fe─N─C CL, Cu/Fe─N─C s‐GDE performed a total FE(C_2+_) of 87.3% with the C_2+_ partial current density of −437.2 mA·cm^−2^ at the applied voltage of 2.89 V, and the value of FE(C_2_H_4_) was up to 46.9%.^[^ ^174^ ^]^

Constructing two catalysts into a tandem catalyst through physical mixing is a facile method.^[^ ^175^ ^]^ Lin et al. adopted a tandem catalyst consisting of Cu_2_O nanocubes combined with Ni SAC (Ni─N─C) at low overpotential with high ethylene selectivity in a vapor‐fed CO_2_ electroreduction system. This is because the CO generated by Ni─N─C increased the local CO coverage near the Cu surface and C─C coupling occurred more readily. FE(C_2_H_4_) reached 45% at −0.6 V versus RHE and the C_2_H_4_ partial current density reached −62 mA·cm^−2^, and the selectivity ratio of C_2_H_4_/CO attained a maximum of 5.5 at −0.7 V versus RHE.^[^ ^176^ ^]^

#### 4.5.3 Other C_2+_ Products

Apart from ethanol and ethylene, few other C_2+_ products have been studied on SACs. Zhao et al. fabricated a Cu SAC (Cu‐SA/NPC), which produced gaseous CO and H_2_ as well as liquid CH_3_COCH_3_, HCOOH, CH_3_COOH, and beyond in a wide range of applied potentials. Interestingly, CH_3_COCH_3_ was the main liquid product, and its FE was 36.7% at −0.36 V versus RHE in an H‐cell with CO_2_‐saturated 0.1 m KHCO_3_ (Figure 8f). To explain this phenomenon, DFT calculation was performed and two structure models including Cu‐pyrrolic‐N_4_ and Cu‐pyridinic‐N_4_ were established. Gibbs free energy of CO_2_‐to‐*COOH and C─C coupling obtained on Cu‐pyrrolic‐N_4_ was lower than that on Cu‐pyridinic‐N_4_ at −0.36 V versus RHE (Figure 8g), indicating the conversion of CO_2_‐to‐CH_3_COCH_3_ was easier to occur on Cu‐pyrrolic‐N_4_. Moreover, the existence of pyridinic‐N_4_ sites improved the stability of intermediates (e.g., *CO, *OCCOH, *CHCOCH_3_) during CO_2_‐to‐CH_3_COCH_3_ and the process of C─C coupling.^[^ ^35c^ ^]^

Recently, Hu et al. constructed a tandem catalyst Ni SACs─Cu NPs, in which the atomically dispersed Ni─N_3_ sites provided the encapsulated Cu sites with sufficient CO coverage for subsequent eCORR. This tandem catalyst realized the conversion of CO_2_‐to‐acetate with FE ≈ 45% at −0.5 V versus RHE in an H‐cell.^[^ ^177^ ^]^ This mode can be considered to be an “adjacent nanostructure strategy”. The adjacent nanostructure of Cu sites and atomically dispersed active sites guarantee that CO produced by atomically dispersed active sites can be efficiently captured by Cu sites for further reduction.^[^ ^178^ ^]^ Besides, Wu et al. designed a two‐step tandem catalytic system that consisted of two electrolysis cells used to convert CO_2_ into CO and then transform CO into C_2+_ products, respectively. The Ni SAC (Ni‐SAG) in the first electrolysis cell successfully obtained an extremely high FE(CO) of 99.2%. Under the atmosphere of CO, the following multi‐hollow Cu_2_O further reduced CO into *n*‐propanol with an FE of 15.9%.^[^ ^179^ ^]^

From the above cases, it is clear to observe the difficulty of obtaining C_2+_ products by adopting a simple SAC for eCO_2_RR. Most of those researchers have used some special methods, including reconstruction of active sites, tandem catalysts, or support effects. In short, the homogeneity of SACs makes them more in need of external forces to assist them in the electroreduction of CO_2_ to C_2+_ products, which can come from other catalysts, supports, or experimental conditions. Due to the achievements of CO_2_‐to‐CO on SACs, it can be seen that the incorporation of SACs into tandem catalysis is the most promising method to obtain C_2+_ products during eCO_2_RR.

## 5 Summary and Outlook

In this review, we critically reviewed the recent development of SACs for eCO_2_RR. SACs exhibit many unique characteristics, such as high product selectivity, high atomic utilization, homogeneous active sites, etc. To ensure the stability of single atoms and inhibit their agglomeration, the strong interaction between atomically dispersed metal atoms and support enables the electron transfer from the support to the center active sites, which is conducive to the activation of extremely stable CO_2_ molecules. This process of electron transfer can be the main source of activity of SACs. Furthermore, due to the homogenous structure of SACs, the geometric and electronic structure of active sites in SACs can be observed by XAS or other characterization methods. Especially, with the rapid development of in situ characterization technologies in recent years, the structural evolution and electronic state changes of the active sites of SACs during the reaction process can be observed by in situ XAS, and the adsorption and desorption behaviors of reactants, intermediates, and products can also be observed by various in situ technologies such as Infrared and Raman spectroscopies. These characterization methods will help researchers explore the real active sites in SACs during eCO_2_RR. However, homogeneous active sites also lead to difficulty in breaking linear scaling relationship and obtaining C_2+_ products, which make the application of SACs limited.

### 5.1 Research Status

To find methods to further improve the performance of CO_2_ conversion, research aimed at the key factors of SACs, including metal atom centers, coordination structure, and electronic properties. As for the metal atom centers, choosing a suitable metal element can make the reaction proceed in the path of one desired product. For example, SACs with *3d* transition metal elements Fe, Co, and Ni tend to produce CO, and those with main group elements In, Sn, and Sb are inclined to produce formate. These SACs usually realize high selectivity for their corresponding products. Especially, Cu SACs have been widely paid attention to due to the ability of other Cu‐based catalysts to produce value‐added C_1_ and C_2+_ products, while this ability also leads to lower selectivity to a certain product. Hence, researchers have attempted to reach high selectivity to those value‐added products with the engagement of SACs.

Based on the flexible and adjustable structure of SACs, many structural modification strategies have been proposed, including regulating coordination number, replacing coordination atom, doping heteroatom, and establishing axial coordination on SACs with the type of M─N_x_─C. When the coordination environment changes, the redistribution of electrons occurs on SACs, changing the adsorption and desorption behavior of reactants, intermediates, and products. This is a common method to regulate the electronic structure of the central metal atom by external factors in SACs.

At present, there are not too many methods to directly regulate electronic properties. Two common ways are to control the valence state of metal atoms during the synthesis process and construct dual atom sites. Generally, due to the strong interaction between supports and center metal active sites, the valence state of the central metal atom does not exist in the highest oxidation state +*n*. Electrons on the supports will transfer to the center metal atoms through the bonds that connect supports to the center metal atoms so that the valance state of the central metal atoms is between 0 and +*n*. A small number of electrons on the center metal atoms can transfer to CO_2_ molecules, improving CO_2_ activation during eCO_2_RR. Therefore, some researchers propose that the central metal atoms with lower valence under electroreduction are the active sites. As for those multivalent metal elements, loading the central metal active sites simultaneously with different oxidation states on the support and then exploring the synergetic effect between them will be another research direction of SACs in the future. Besides, the dual atom site strategy which introduces another neighboring metal atom will promote electron redistribution and break the limitation of the traditional SACs to provide a synergetic effect. This strategy still maintains the advantages of high atomic utilization in SACs and enables the improvement of the adsorption behavior of key intermediates and catalytic reaction routine. It is worth mentioning that Cu dual atom sites possess the opportunity to achieve the conversion from CO_2_ to C_2+_ products，simultaneously.

So far, the supports of SACs for eCO_2_RR are still dominated by carbon substrates which have various morphologies such as nanotubes, nanosheets, and carbon spheres, featured with high specific surface area and high conductivity. On one hand, carbon substrates are easily loaded with atomically dispersed active sites and realize efficient CO_2_ adsorption. On the other hand, rich electrons on carbon substrates can transfer to center metal atoms faster to promote CO_2_ activation. MOF (or other organic frameworks) can conveniently and quickly anchor and separate metal ions during the synthesis process to avoid atom agglomeration. However, a large number of MOF‐based SACs for eCO_2_RR currently available still choose ZIF‐8 as the precursor. Broadening the choice of MOF precursors is one of the keys to widening the applications of SACs. A small number of atomically dispersed atoms are anchored on the metal substrate to form SAA, while the real active sites of SAA have not been distinguished yet. In addition, a few oxide‐based SACs provide an idea for the development of more efficient SACs in the future: more consideration should be given to the synergetic effect between active sites and supports, and the support effects should be used to assist atomically dispersed active sites in reducing CO_2_ to value‐added products (CH_4_ or other C_2+_ products).

The products of eCO_2_RR on SACs are mainly CO and formate, and few studies have successfully generated CH_3_OH, CH_4_, or C_2+_ products. For CO and formate which only require a two‐electron transfer step, researchers have focused more attention on improving CO_2_ capture and current density or modifying the synthesis method and working conditions. For CH_3_OH, CH_4_, and C_2+_ products, which involve multiple‐electron transfer steps, atomically dispersed active sites have become the disadvantage of SACs in that ordinary SACs cannot complete the complex reaction process.

### 5.2 Remaining Challenges And Outlooks

For the eCO_2_RR process, there are three important indicators: selectivity, current density, and stability. How to achieve higher achievements in these areas remains a huge challenge.

As for high selectivity, SACs attain high selectivity for those simple C_1_ products (CO, formate) due to their high atomic utilization and homogenous active sites. However, competitive HER makes it difficult for SACs to achieve a high selectivity at higher potential or current density. However, due to their uniform active sites, it is difficult to achieve other deep products (CH_3_OH, CH_4_, and C_2+_ products). How to design SACs and achieve selectivity for complex products is a principal issue. As an example, CH_3_OH and CH_4_ are a group of competing products from further electroreduction of *CO intermediates, and the key difference lies in whether a subsequent dehydration process occurs. Therefore, one can try to differentiate by choosing supports with different oxophilicity properties. On the supports with stronger oxophilicity, the oxygen atoms in the *CO intermediate will tend to stay on the supports to induce the electroreduction of *CO to CH_4_. Conversely, on the supports with weaker oxophilicity, *CO may be reduced to CH_3_OH rather than CH_4_. Meanwhile, the supports themselves can also act as *H‐producing site for the subsequent *CO electroreduction to other products by providing additional *H intermediates. It is also possible to use the supports to wrap some metal nanoparticles as a method to increase the current density of SACs in the catalytic process. For C_2+_ products, tandem catalysis can divide a complex one‐step reaction into a two‐step reaction, disassembling the original CO_2_ → C_2+_ step into CO_2_ → CO and CO → C_2+_ sequences. This strategy can effectively avoid the excessively low generation efficiency of C_2+_ products caused by the direct desorption of CO after the reduction of CO_2_ to CO on Cu‐based electrocatalysts. The products generated in the previous step in the tandem catalyst are used as reactants in the next step to maximize the generation efficiency of C_2+_ products. The high *CO coverage originating from the CO generation catalyst is also more favorable for the C─C coupling step. With the emergence of the tandem strategy, it has become a facile and efficient method to take SACs as one part of the catalysis system to reduce CO_2_ to CO, and then CO can be transferred to another part of the catalysis system to undergo further reduction to value‐added products. This method can not only keep the high CO selectivity of SACs but also make up for the difficulty of multi‐electron transfer reactions caused by homogenous active sites in SACs during eCO_2_RR.

In terms of current density, low metal atom loading makes the current density in the H‐cell usually between −10 – −30 mA·cm^−2^. Therefore, it is necessary to use higher‐level electrolytic cells to enhance the current density of the reaction process. In a simple H‐cell, due to the small working area and low solubility of CO_2_ in water, the electroreduction of CO_2_ is restricted because of mass transfer. Meanwhile, during eCO_2_RR process, as the pH of the aqueous solution increases, the reaction will be more favorable. For a closed H‐cell, introducing a large amount of CO_2_ into the alkaline electrolyte will lead to a reaction between CO_2_ and the electrolyte. Therefore, only neutral electrolytes can be used in the H‐cell, which reduces the current density during the reaction process. To address the shortcomings of this reactor, researchers applied eCO_2_RR to flow‐cell and MEA. In the flowing phase electrolysis cell, the incoming flowing CO_2_ and cathode electrolyte are divided by the catalyst layer coated on the gas diffusion layer (GDL), and CO_2_ will diffuse to the catalyst surface through GDL to form a three‐phase interface for reaction. This reaction mode separates the contact between the gas and liquid phases. Therefore, KOH solution can be used as the electrolyte in flow‐cell to replace KHCO_3_ solution, improving the current density during the reaction, usually reaching a value of over −100 mA·cm^−2^, meeting industrial grade requirements. Nowadays, it is currently necessary to use a flow‐cell for eCO_2_RR. Furthermore, the flow‐cell also faces a series of problems such as carbonate deposition, making it necessary to adopt a zero‐gap design MEA. In MEA, the cathode does not require electrolyte, but only humid CO_2_, and the OH^−^ in the electrolyte on the anode side will be transferred to the cathode side through the anion exchange membrane to participate in the reaction.

When it comes to long‐term stability, researchers should focus on the catalyst itself and optimize the design and synthesis of materials. From this review, it is not difficult to find that the SACs used in eCO_2_RR are composed of carbon materials, involving a large number of high‐temperature calcination processes, which will cause a large number of single‐atom active sites to aggregate into particles, reducing catalytic reaction activity. Therefore, based on the current design, more synthesis methods for SACs should be developed and other materials could be explored as supports. For example, currently, there are only a small number of SACs based on organic frameworks. researchers can take advantage of organic frameworks to anchor metal sites, as mentioned earlier, to construct single or dual atom sites within organic frameworks to promote the electroreduction from CO_2_ to value‐added products. Meanwhile, organic frameworks have advantages such as structural stability and a large number of modification methods. In addition to the above design ideas, it should also be considered whether the catalyst can withstand higher applied potential. Nowadays, most catalysts achieve excellent product selectivity at lower applied voltages, but under high applied voltages accompanied by high current density, the reaction is likely to switch from eCO_2_RR to HER. How to achieve high product selectivity under high applied voltage and high current density to meet industrial demands is also a challenge that researchers must address.

In short, in the design and synthesis of SACs in the future, we should break the original design idea that only the center metal atoms act as the only active sites in the process of eCO_2_RR. When SACs individually cannot meet our demands to produce value‐added products, externally assisted forces must be applied, such as synergistic strategy and tandem strategy. As SACs are gradually applied in industrial production, they will have a favorable impact on the industrial energy structure and the earth's ecological environment. From an energy point of view, CO_2_ can be used as a feedstock for a variety of fuels and industrial production necessities to improve the current production models. From the perspective of the ecological environment, it can help to curb excessive CO_2_ emissions to promote sustainable development of the Earth. We believe that SACs will broaden applications in the future field of eCO_2_RR.

## Conflict of Interest

The author declares no conflicts of interest.
